# OCT-based diagnosis of glaucoma and glaucoma stages using explainable machine learning

**DOI:** 10.1038/s41598-025-87219-w

**Published:** 2025-01-28

**Authors:** Md Mahmudul Hasan, Jack Phu, Henrietta Wang, Arcot Sowmya, Michael Kalloniatis, Erik Meijering

**Affiliations:** 1 School of Computer Science and Engineering, University of New South Wales, Sydney, NSW Australia; 2 School of Optometry and Vision Science, University of New South Wales, Sydney, NSW Australia; 3 Centre for Eye Health, University of New South Wales, Sydney, NSW Australia; 4 Faculty of Medicine and Health, University of Sydney, Camperdown, NSW Australia; 5 School of Medicine (Optometry), Deakin University, Waurn Ponds, VIC, Australia; 6 University of Houston College of Optometry, University of Houston, Houston, TX USA

**Keywords:** Optical coherence tomography, Glaucoma, Perimetry, Explainable machine learning, SHAP analysis, Partial dependency analysis, Optic nerve diseases, Biomedical engineering, Computer science

## Abstract

Glaucoma poses a growing health challenge projected to escalate in the coming decades. However, current automated diagnostic approaches on Glaucoma diagnosis solely rely on black-box deep learning models, lacking explainability and trustworthiness. To address the issue, this study uses optical coherence tomography (OCT) images to develop an explainable artificial intelligence (XAI) tool for diagnosing and staging glaucoma, with a focus on its clinical applicability. A total of 334 normal and 268 glaucomatous eyes (86 early, 72 moderate, 110 advanced) were included, signal processing theory was employed, and model interpretability was rigorously evaluated. Leveraging SHapley Additive exPlanations (SHAP)-based global feature ranking and partial dependency analysis (PDA) estimated decision boundary cut-offs on machine learning (ML) models, a novel algorithm was developed to implement an XAI tool. Using the selected features, ML models produce an AUC of 0.96 (95% CI: 0.95–0.98), 0.98 (95% CI: 0.96–1.00) and 1.00 (95% CI: 1.00–1.00) respectively on differentiating early, moderate and advanced glaucoma patients. Overall, machine outperformed clinicians in the early stage and overall glaucoma diagnosis with 10.4 –11.2% higher accuracy. The developed user-friendly XAI software tool shows potential as a valuable tool for eye care practitioners, offering transparent and interpretable insights to improve decision-making.

## Introduction

Glaucoma, also known as glaucomatous optic neuropathy, encompasses a series of optic nerve diseases causing retinal ganglion cell impairment and death. In developed nations, it ranks as the second most common cause of irreversible blindness^1^. Early detection and treatment of glaucoma are crucial as the damage it causes is permanent and irreversible. A meta-analysis study suggests that the number of individuals diagnosed with glaucoma is projected to increase significantly over the next two decades, almost doubling from 76 million in 2020 to an estimated 112 million in 2040^2^.

Currently, the diagnosis and management of glaucoma requires a comprehensive examination including tonometry^3^, visual field (VF) testing^4,5^ and fundoscopy^6^, within a routine ophthalmic assessment that aims to rule out other potential causes of optic nerve disease. Furthermore, ocular imaging, such as optical coherence tomography (OCT), has become a clinical standard for glaucoma assessment^7^. Although many of these tests can be competently conducted by trained technicians, the interpretation of the results requires clinical expertise for personalised care^8^ which therefore places a strain on the limited resources of the healthcare system.

The increase in the number of patients puts further pressure on healthcare systems and makes it difficult to diagnose and treat glaucoma in a timely manner using manual methods^9,10^. Reus et al.^11^ reported clinician variability in glaucoma diagnosis in 11 different counties (Austria, Belgium, Finland, France, Germany, Greece, Hungary, Italy, the Netherlands, Spain and the United Kingdom) based on the assessment of stereoscopic optic disc images, and found a significant difference between clinicians’ performance across countries (p < 0.0005). Based on the overall performance of clinicians on 243 total observations, the clinicians’ mean sensitivity was 74.7% (range 43.8–100%), specificity was 87.4% (25–100%) and accuracy was 80.5% (61.4–94.3%). This indicates that the human error rate can vary significantly between clinicians and result in misdiagnosis.

Due to the known variability across clinicians and in light of the above findings, there is emerging evidence that computer-aided methods may assist in improving the objectivity, efficiency and accuracy of glaucoma diagnosis, in a more accessible and less expensive manner.^12^ A recent review of artificial intelligence (AI)-based studies on glaucoma diagnosis revealed that most of them use colour fundus images from a public database and deep learning (DL) methods.^12^ While DL applied to fundus images can provide the user with an end-to-end diagnostic framework, such black-box^13^ approaches entail huge computational costs and hardware resources^14^, which may raise economic and environmental concerns^15^. Also, those approaches require huge datasets for training purposes, which has led some studies to include self-reported glaucoma patients^16^ and glaucoma suspects^17,18^ in the glaucoma group from public datasets, such as the UK biobank^16^. As such, those approaches have limited reliability, which hinders real-life clinical applicability. A traditional machine learning (ML) approach trained on a more reliable dataset of OCT images and producing more explainable predictions may better serve clinicians.

The existing handful of OCT-based studies using ML leave much room for improvement. First, most methods have focused on spatial domain features (extracted directly from images) and particular types of analysis, such as retinal nerve fibre layer (RNFL) thickness analysis^19,20^, but missed the frequency-domain patterns that can be extracted from the images and other changes in the retinal ganglion cell (GC) and macular (MC) area. Second, most studies did not perform any feature selection while diagnosing glaucoma^21–26^, leading to a redundant number of features supplied to the ML models^21,23^. Third, the focus of most studies on ML for diagnosing glaucoma has been on classifying clearly glaucomatous and normal eyes. However, it is important to identify the different severity levels of glaucoma, especially in the early stages and to track disease progression^27^. Fourth, past studies did not provide possible explanations or interpretations but used black-box AI models focusing only on performance metrics. Lastly, past studies were typically based on experimental Python or MATLAB code, and there is a scarcity of studies developing and releasing user-friendly AI apps for real-time diagnosis of glaucoma^12^.

In this study, we leverage spatial domain features, such as the RNFL temporal-superior-nasal-inferior-temporal (TSNIT) pattern, to extract frequency domain information^28–30^, aggregate GC inner plexiform layer (IPL) and MC thickness features, apply feature selection techniques, and assess the performance of ML models in diagnosing glaucoma stages for early, moderate and advanced glaucoma. Furthermore, we perform a sub-analysis to classify advanced glaucoma based on mean deviation (MD) and central field defects. The main contribution of this study is the use of explainable AI (XAI) techniques to uncover the black-box ML model to improve understanding, trustworthiness and reliability. To the best of our knowledge, this is the first study to include spatial domain RNFL, GC-IPL and MC thickness and frequency domain TSNIT features within an XAI framework, specifically SHapley Additive exPlanations (SHAP) analysis and partial dependency analysis (PDA), for OCT-based diagnosis of glaucoma. Leveraging the proposed XAI techniques, we also present a handy software tool to assist clinicians in glaucoma diagnosis.

## Materials and methods

### Data collection and categorisation

#### Ethics statement

This was a cross-sectional study using prospectively acquired data from the files of patients seen by the Centre for Eye Health (CFEH), University of New South Wales (UNSW), Sydney, Australia. Ethics approval was provided by the Human Research Ethics Committee of UNSW (HC210563). The study adhered to the tenets of the Declaration of Helsinki. All subjects provided their written informed consent for use of their de-identified clinical data for research purposes.

#### Data acquisition

The study collected data from patients seen between 2015 and 2021 at CFEH. The clinic is a referral-only service for patients with visual pathway diseases, including glaucoma. The glaucoma examination protocols of CFEH have been described previously^31–34^. In summary, the glaucoma examination protocols used at the clinic include a comprehensive assessment of the patient’s history, visual acuities, anterior segment examination, applanation tonometry, pachymetry, gonioscopy, dilated stereoscopic examination of the optic nerve head and the macula, standard automated perimetry, colour fundus photography of the optic disc and posterior pole and OCT imaging. The study excluded patients classified as "glaucoma suspect," and those with non-glaucomatous optic atrophy. As per the clinical protocols of CFEH^34^, the diagnoses were made by examining clinicians (experienced optometrists and ophthalmologists) and if required, reviewed remotely by senior clinicians, with further examination by a third expert for inclusion in the study. While phenotyping, any images with artefacts were flagged by the clinicians.

#### Data categorisation

We categorised the ocular diagnoses of eyes from glaucoma patients within the present cohort into three categories, based on the mean deviation of Visual Fields (VF). More specifically, we used the criteria as per Mills et al.^35^ to determine glaucoma severity levels (early glaucoma as MD up to -6 dB, moderate as -12 dB < MD ≤ -6 dB, and advanced as MD ≤ -12 dB) and our categories were healthy (n = 334 eyes); early glaucoma (n = 86 eyes); moderate glaucoma (n = 72 eyes); advanced glaucoma based on MD (n = 37 eyes); and advanced glaucoma based on central field defect (CFD)^36^ (n = 73 eyes). The advanced stage of glaucoma was defined on MD. The presence of a CFD that did not meet the MD criterion was not deemed as advanced. Overall, the group of consisted of 334 normal eyes and 268 glaucoma eyes. Normal individuals were matched to the ages of the glaucoma patients (Table 1). The reference labels determined by these criteria were also used for training the ML models (described in Table 1).

**Table 1:** Demographics of subjects included in this study.

Glaucoma types	N	Age (mean ± std)	RE	LE	RE + LE
Normal	167	67.77 ± 6.85	167	167	334
Early	86	66.34 ± 8.86	48	38	86
Moderate	72	65.90 ± 11.68	36	36	72
Advanced (based on MD)	37	64.70 ± 10.83	24	13	37
Advanced (based on CFD)	73	61.78 ± 13.39	39	34	73
Glaucoma (total)	268	64.48 ± 11.44	147	121	268

*N* Number of patients, *RE* right eye, *LE* left eye, *std* Standard deviation, *MD* mean deviation, *CFD* central field defect.

### Feature extraction

#### Extraction of spatial domain features

**Fig. 1:**
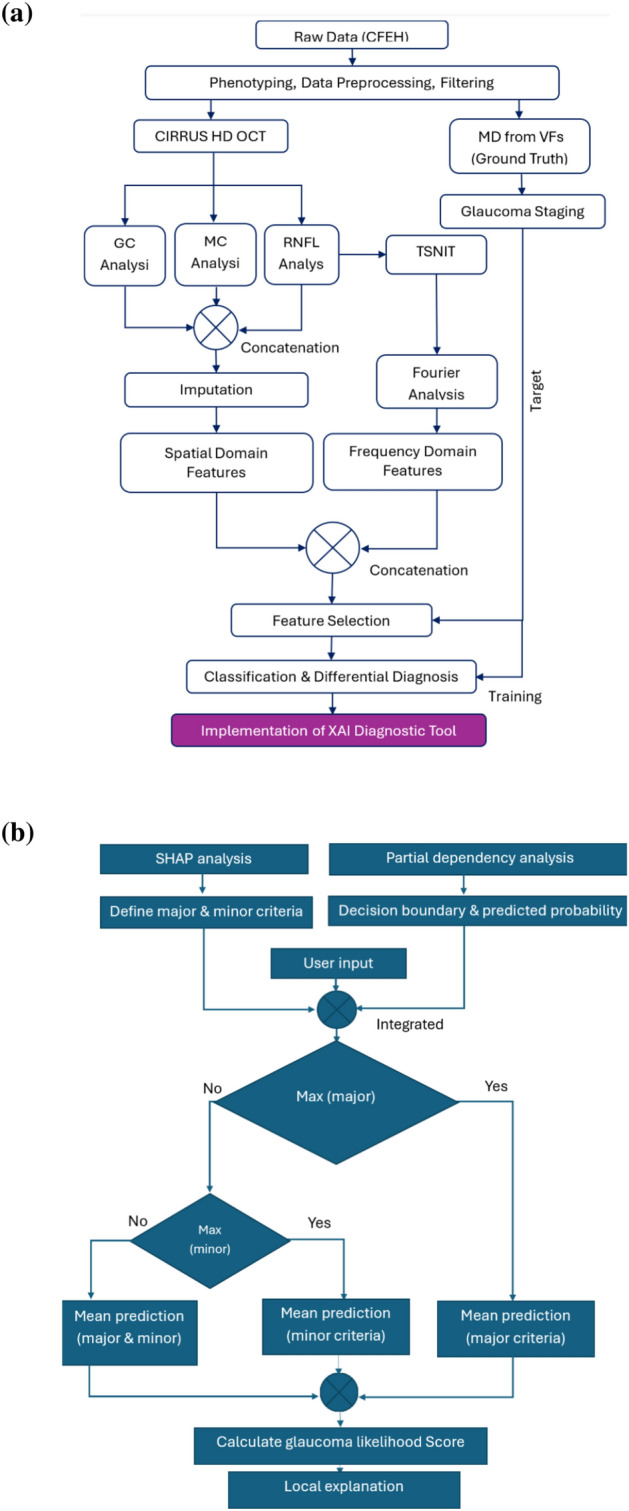
(**a**) Experimental procedure of this study. The key steps include raw data phenotyping, feature extraction in the spatial and frequency domain, feature selection, classification and model interpretation using XAI. (**b**) Flow-chart of the implementation of the explainable diagnostic tool based on SHAP and PDA (Excel tool). Blue shaded boxes represent explainable analysis. Note: CFEH: Centre for Eye Health, GC: Ganglion Cell, MC: Macular, RNFL: Retinal Nerve Fibre Layer, TSNIT: Temporal-Superior-Nasal-Inferior-Temporal, MD: Mean Deviation.

The spatial domain features, e.g., RNFL, GC-IPL and macular thickness, provide information about the distribution, thickness and variations of the specific retinal layers. Features from these scanning protocols are known to be useful for distinguishing between healthy, glaucoma suspect and glaucomatous eyes to various degrees and with levels of inter-parameter correlation^37^. These features refer to the characteristics and patterns observed within the two-dimensional space of the OCT image itself. Using the CIRRUS HD-OCT software (ZEISS), all the spatial domain features were extracted as numerical data in this study (Fig. 1(a)). The following analyses were performed with the CIRRUS software: optic nerve head (ONH) and RNFL *oculus uterque* (both eyes) (OU) Analysis, Ganglion Cell OU Analysis, and Macular Thickness OU Analysis (Supplementary Fig. 1; Supplementary Table 1). The software was used to extract the 256-point TSNIT patterns, plot them in the spatial domain and compute statistical features such as mean, median, standard deviation, skewness and kurtosis of the whole TSNIT (Supplementary Fig. 2), and each of the temporal, superior, nasal and inferior quadrants. Additionally, some information theory-based features were also derived from the TSNIT data, i.e., Shannon entropy, Fisher information and signal-to-noise ratio. To observe the effect of these features on visual fields, the structural–functional relationship was plotted (Supplementary Fig. 3).

#### Derivation of frequency domain features

The 256-RNFL values extracted from the RNFL thickness analysis form the TSNIT pattern, which is a typical 'double hump pattern’^30^, representing the superior and inferior in the first and second hump respectively. In line with Essok et al.^30^, we applied the fast Fourier transform (FFT) to the OCT TSNIT pattern to transform the spatial domain into the frequency domain and extracted several frequency-domain features, including the power spectral density (PSD) of the TSNIT pattern (overall and RNFL quadrants) using the ‘Welch’ and ‘FFT’ method, the PSD of the TSNIT pattern after discrete wavelet transformation (DWT) (levels 1 to 4), the slopes of the harmonics of the PSD, and the spectral entropy (Supplementary Table 1).

#### Feature imputation

For some patients, the data contained artefacts such as image truncation, inaccurate delineation of the cup and/or disc margin, media opacities and segmentation error, affecting either RNFL, GC-IPL or MC measurements. We included any patients having at least one artefact-free parameter (e.g., RNFL). The parameters containing artefacts were considered as missing data, amounting to 3.65% of the total samples (e.g., GC-IPL), and multiple imputation using chained equations (MICE)^38^ imputation was performed to impute the missing data points based on other existing artefact-free data (e.g., RNFL & MC). MICE is a statistically robust imputation technique that preserves the inherent relationships between features for a specific patient^39,40^. Unlike single imputation methods, MICE generates multiple plausible datasets while accounting for the uncertainty associated with missing features (e.g., RNFL) by leveraging information from other available features^40^ (e.g., GC-IPL and MC). Patients with at least one artefact-free parameter were included, and artefact-affected parameters were treated as missing data. MICE imputation used the existing artefact-free data to reconstruct these values. However, when all the RNFL, GC-IPL or Macular thickness data were affected by artefacts, the sample was removed from the dataset.

#### Feature selection

Eliminating irrelevant features is critical as they can hinder the accuracy of classification models^41^. To address this problem in our study, a feature selection process was carried out on the spatial-frequency domain features derived from the OCT-based analysis. Based on the literature, filter methods in feature selection are independent of any learning algorithms and are effective in various real-world datasets^42–44^. Given the computational complexity of feature selection and previous studies on the subject^42,43^, this study concurrently employed two univariate filter-based feature selection techniques: analysis of variance (ANOVA) F Test and Pearson’s correlation-based feature selection. The ANOVA F-test was chosen because it captures inter-class variability by identifying features with high variation between group means relative to class labels^45,46^. As a univariate feature selection method, it evaluates each feature independently, making it an effective base for ranking features with higher discriminative power. Pearson’s correlation-based method operates on the hypothesis that a feature is valuable if it demonstrates relatively low correlation with other features^46,47^. In this process, highly correlated features (r > 0.98) were identified using Pearson’s correlation, retaining only the top-ranking feature with the highest F-score from each group of correlated features. The remaining features were ranked by their F-scores, and the ‘SelectKBest’ scikit-learn module^48^ was applied, selecting the K highest scored features with the wrapper approach^49^. In this approach, a tuned classification model with five-fold cross-validation is used to determine the optimal cut-off by observing validation accuracy with an increasing number of top-ranking features from the spatial-frequency domains.

### Classification models

To date, there is no standardised or generalised model for the classification of glaucoma stages. Due to the distinct computational cost and complexity^50,51^ and based on current literature, three supervised learning models were employed for classification: K-Nearest Neighbour (KNN), Support Vector Machines (SVM), and Random Forests (RF) classifier. The hyperparameters of each classifier were adjusted using an iterative process to obtain the best accuracy after cross-validation (Supplementary Table 2). For the KNN classifier, different values of 'k' (range: $1\le k \le 40$) were used to tune the model, and the 'k' value that corresponded to the best training accuracy was selected (optimal value of k = 7 for overall glaucoma vs normal). For SVM, the Gaussian radial basis function (RBF) kernel was utilised, and the 'C' (optimal value: 10 for overall glaucoma diagnosis) and 'γ' (optimal value: 0.01 for overall glaucoma diagnosis) parameters were tuned using the grid-search algorithm to achieve optimal parameter values for best accuracy after cross-validation. For the RF classifier, the number of trees in the forest (‘n_estimate’) (optimal value: 100 for overall glaucoma) and the maximum depth of the tree ('max depth’) (optimal value: 3 for overall glaucoma) were tuned through a grid-search algorithm. Feature scaling was performed before supplying the features to the KNN and SVM models (min–max scaling for KNN and standardisation for SVM^52^); however, we did not perform any feature scaling for RF as it does not require scaling given the nature of the algorithm^52^, which further helped us explain the features using XAI.

In our dataset, disease-positive cases (i.e., the number of glaucomatous eyes) are usually less frequent than normal or healthy cases. In medical diagnosis, the cost of misclassifying a disease-positive case is typically much higher than misclassifying a healthy case. To address this, the synthetic minority over-sampling technique (SMOTE)^53^ was used in our study to overcome data imbalance (imbalance ratio for glaucoma : normal = 0.802 : 1). SMOTE was selected because it generates synthetic samples by interpolating between minority class samples rather than simply duplicating existing data, which helps to reduce the risk of overfitting^54^. This approach effectively increases the representation of the minority class (glaucoma in our case) while preserving variability within the dataset^53,54^. It has been widely used in retinal disease diagnostic studies, including retinal image diagnosis, like glaucoma^55,56^ and age-related macular degeneration^57^ and preeclampsia^57^.

We note that SMOTE and MICE-based augmented data were not used in the feature selection and explainable analysis. Additionally, we ran a sub-analysis with all the classification models using artefact-free data only (without MICE-based imputation and SMOTE-based oversampling) to maintain clinical transparency and reliability of the results. All models were implemented, trained and tested using Python version 3.7 in the Google Colab^58^ platform. Performance evaluation, patient-level splitting and cross-validation have been described in supplementary methods.

### Explainable machine learning

#### Shapley additive analysis

SHapley Additive exPlanations (SHAP) is a method for explaining the output of ML models by attributing the prediction to the features that contributed to it^59^. It does this by using the concept of Shapley values from game theory, for fairly distributing the “credit” for a prediction among the contributing features. Shapley values are useful to calculate the importance of each feature in a model’s prediction and have been used to interpret ML models in medical^60^ and other domains^61,62^. Generally, the Shapley value of a feature *i*, denoted ${\varphi }_{i}\left(val\right)$, indicates the contribution of a feature value ($val$) in a payout. It sums over all possible feature combinations ($S$) and measures the difference in feature values ($val\left(S\cup [i]\right)-val(S)$), adjusting for the size of the combinations^63^ (Eq. 1).

1 $$\varphi_{i} \left( {val} \right) = \sum_{S \subseteq [1, \ldots ,m]\backslash [i]} \frac{{|S|!\left( {m - |S| - 1} \right)!}}{m!}\left( {val\left( {S \cup \left[ i \right]} \right) - val\left( S \right)} \right)$$

Here, *i* is the feature of interest, *m* represents the total number of features excluding the feature *i*, while $S$ is the subset of the features excluding feature *i*, $val\left(S\cup [i]\right)$ represents the total value generated by the coalition that includes feature *i*, and $val(S)$ represents the value of the coalition *S*, which is the total value generated by the coalition that does not include feature* i*.

While Shapley values are a concept from cooperative game theory, SHAP is a unified framework that generalises Shapley values and typically refers to the aggregated ‘Shapley values’ as ‘SHAP values’^63^. This study used SHAP-based feature importance, SHAP dependence plot and SHAP interaction plot as a part of the SHAP analysis, both in global and local scopes^63^.

#### Partial dependency analysis

Partial dependency analysis (PDA) is another technique used for interpreting and explaining the output of ML models^64^. PDA helps to understand the relationship between a single feature in relation to the model’s output, holding all other features constant. The resulting plot from PDA representing the relationship between feature values and the model’s output is called a partial dependency plot (PDP). PDPs help understand the marginal effect of the features on the predicted probability of the ML models and have been widely used in interpreting real-world problems^61,62^, specifically for identifying the decision boundary^65–67^ in ML-based classification problems. The partial dependence function^63^ (Eq. 2) is defined as:

2 $$\hat{f}_{i} \left( i \right) = E_{S} \left[ {\hat{f}_{i} \left( {i, S} \right)} \right] = \hat{f}_{i} \left( {i, S} \right)dP\left( S \right)$$

Here*,* $i$ is the feature of interest, for which we want to know the effect on the prediction, ${\widehat{f}}_{i}\left(i\right)$ represents the predicted outcome or response variable for the specific feature *i*, based on a ML model, *S* is the set of the other features. ${E}_{S}$ stands for expected or average value of the function ${\widehat{f}}_{i}\left(i, S\right)$ over all possible values of the set of features $S$. ${\widehat{f}}_{i}\left(i, S\right)$ is the predicted outcome or response variable for *i*, based on a ML model, which explicitly indicates the dependence of the feature of interest *i* on the set of other features S. $d{\mathbb{P}}(S)$ represents the probability density function (PDF) / probability distribution over the set of features $S$.

The partial function is approximated using the Monte Carlo method^63^, wherein averages are computed based on the training data (Eq. 3):

3 $${\widehat{f}}_{i}\left(i\right)= \frac{1}{n}\sum_{j=1}^{n}{\widehat{f}}_{i}\left(i, {S}^{(j)}\right)$$

Here, ${\widehat{f}}_{i}\left(i\right)$ is the average marginal effect on the prediction / partial dependence function for feature of interest (*i*), ${S}^{(j)}$ is the set of all possible combinations of the other features in the ML model, in which we are not interested, where* j* ranges from 1 to *n* (the number of samples in the dataset).

#### Diagnostic tool

Based on the SHAP-based global feature importance and PDA-based predicted probabilities for each feature, we developed two software tools to assist clinicians to calculate the glaucoma likelihood of patients based on basic CIRRUS OCT data. The first one is an Excel (Microsoft 365 version-2019) based tool, where the clinicians can input relevant features, categorised into a major and a minor group. At the backend, the PDA-based predicted probabilities and estimated feature cut-offs at the decision boundary were used to make a decision based on the outcome defined with major and minor criteria (Fig. 1(b)). If the maximum of the major criteria is fulfilled, then the glaucoma likelihood score is based on the major criteria. Otherwise, the algorithm checks for the maximum minor criteria to be satisfied to produce a likelihood based on the minor criteria. If the second condition is not fulfilled, the likelihood score is calculated as the mean of major and minor criteria altogether.

The second tool is a web-based app, where a RF model has been deployed. This tool uses the most important features based on SHAP analysis; however, the key difference from the Excel app is that here we let the model decide on using the features based on the RF model rather than defining major and minor criteria and weighted likelihood scores. To validate the likelihood score produced by the apps, the glaucoma likelihood scores (Excel app) and predicted probabilities (Web app) were plotted corresponding to the MD values of each sample (excluding the advanced glaucoma cases based upon central VF loss). The web app was developed using the Flask framework and a Python web framework, and incorporates HTML templates for the user interface. It follows a client–server architecture, where the client interacts with the web application through a web browser, and the server handles the requests and provides responses.

We developed two distinct sets of machine learning models: non-explainable models (leveraging both spatial and frequency domain features) and explainable models (utilising a small set of explainable spatial domain features). The initial non-explainable models were built using features derived from RNFL, GC-IPL, and MC thickness in the spatial domain, as well as the frequency domain features. These models (the white-shaded boxes in Fig. 1a) are best suited for integration with OCT machine software (e.g., CIRRUS HD-OCT). Here, the availability of 256 TSNIT data points enables the derivation of frequency domain components, which can be concatenated with spatial domain features to enhance glaucoma diagnosis.

In contrast, the explainable models are specifically designed for deployment on edge devices (e.g., mobile phones, tablets, or web apps) that are not directly connected to the OCT machine or its software. In this scenario, the 256 TSNIT data points required to compute frequency domain features are unavailable. Instead, the spatial domain features represent global measures derived from the TSNIT data points (e.g., RNFL superior, nasal, inferior, and temporal thicknesses are calculated as the mean of the first 64, second 64, third 64, and fourth 64 data points, respectively). Additionally, the clinical interpretability of frequency domain features is limited due to the lack of studies exploring their significance. To ensure practical utility and alignment with the needs of clinicians, the explainable models were re-trained exclusively with spatial domain features only.

## Results

### TSNIT plots and statistical analysis

A TSNIT plot was constructed showing the spatial domain TSNIT pattern for early, moderate and advanced glaucoma for both right (Supplementary Fig. 2 (a)) and left (Supplementary Fig. 2 (b)) eyes separately. The plots indicate that for both left and right eyes, the RNFL thickness value in the inferior and superior quadrant is significantly reduced (p < 0.05) when a patient progresses from early to moderate and finally advanced glaucoma. Additionally, scatter plots of the RNFL/GC-IPL thickness versus MD of visual fields to visualise the structural–functional relationship (Supplementary Fig. 2) were similar to previous published works^68^.

### Selected features

Feature selection was performed on the 67 spatial and 64 frequency domain features (total features: 131) extracted from the OCT-based analysis (Supplementary Table 1). Feature selection was performed concurrently using ANOVA F Test and Pearson’s correlation method (see *Feature Selection* in *Materials and Methods*). Highly correlated features were excluded (r > 0.98) retaining the best one having highest F-score as some of the features within the spatial domain, frequency domain, or cross-domain had higher correlation between themselves. Including these highly correlated features would not add information, while increasing redundancy of the feature set, adding complexity to the model’s prediction and reducing interpretability. For example, within the spatial domain features, ILM-RPE thickness central subfield thickness has a higher correlation with ILM-RPE centre-foveal thickness (r = 0.986). Additionally, ILM-RPE thickness-volumetric cube and ILM-RPE thickness-average cube are highly correlated (R = 0.998). As all those four features are correlated, only ILM-RPE thickness-average cube was retained among the four, as it has the highest F-score among the correlated features.

**Fig. 2:**
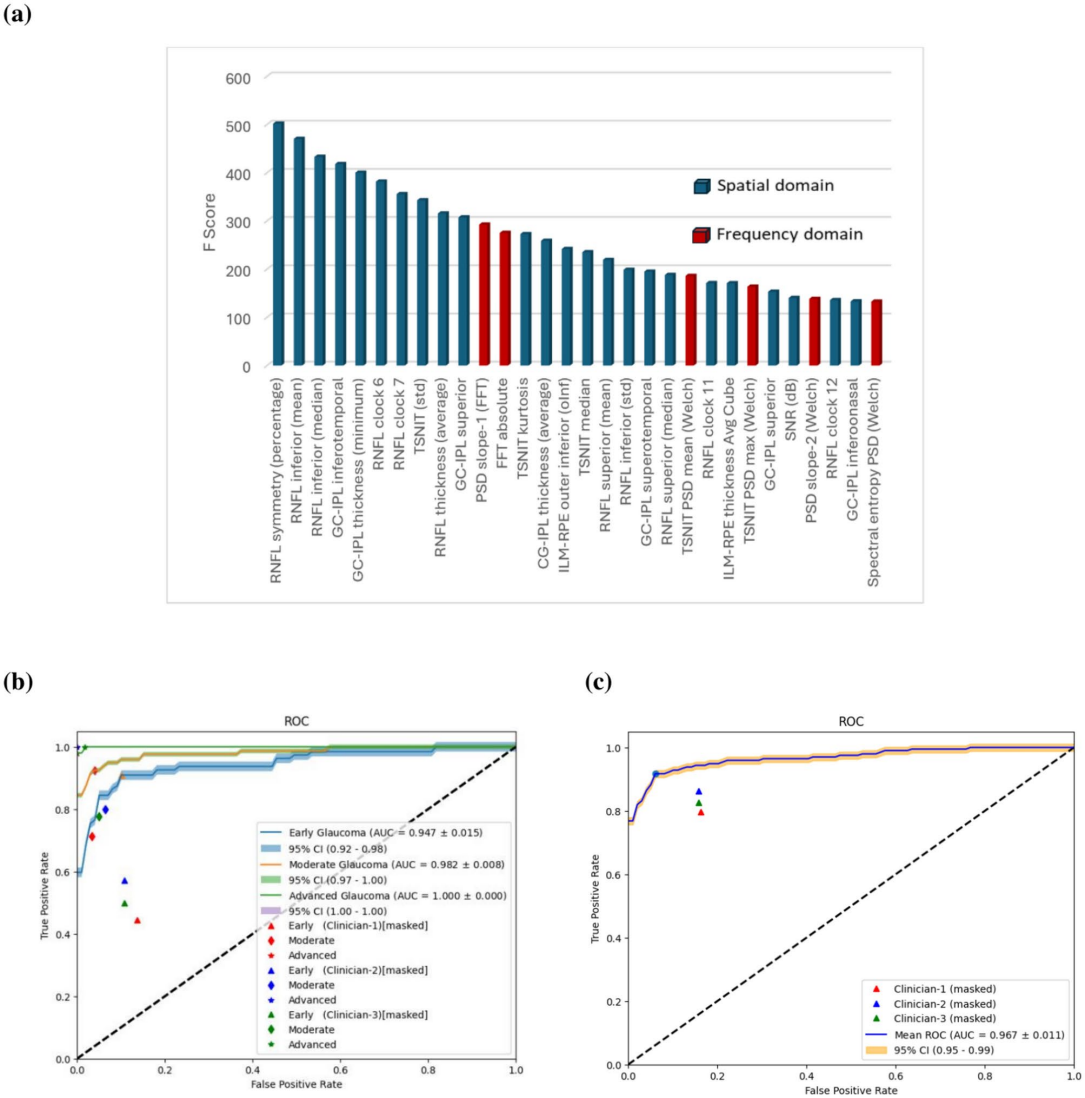
(**a**) Illustration of top 30 features selected by feature selection techniques. (**b**) Human versus Machine (RF) performance comparison for Glaucoma stage diagnosis. (**c**) Overall glaucoma diagnosis. Two optometrists and one glaucoma specialist were provided with RNFL and GC-IPL thickness data with masking (only keeping the numerical thickness values that were fed to the ML models). All the normal and glaucoma patients were randomly assigned, so the clinicians could not predict the order in which they appeared. Once they completed the grading, sensitivity and specificity were calculated (from TP, FP, TN, FN) and plotted in the ROC plot generated for the RF model (using the same data with one-vs one approach) for comparison. Reported AUC = mean ± standard error, standard error = standard deviation/ √n , n = 5 (for five-fold cross validation). Note: RNFL: Retinal nerve fibre layer, GC-IPL: ganglion cell–inner plexiform layer, ILM-RPE: Inner limiting membrane-retinal pigment epithelium, TSNIT: temporal-superior-nasal-inferior-temporal, std: standard deviation.

Once the highly correlated features were excluded (keeping the topmost with highest F-score), the K-highest ranked features were selected to observe the validation accuracy wrapping with an RF classifier (see *Feature Selection* in *Materials and Methods*). Adding features with F-scores below 30 did not contribute to improved validation accuracy; consequently, a cut-off F-score of 30 was established, corresponding to approximately 6% of the highest-ranked feature’s F-score (502). This resulted in the selection of 66 features (45 spatial domain and 21 frequency domain) out of the initial 131 features (Supplementary Table 3), representing nearly 50% of the total features. The feature ranking shows that the topmost features are obtained from the RNFL analysis, with RNFL symmetry and RNFL inferior having the highest ANOVA F score (Fig. 2 (a). Among the frequency domain features, PSD values at the inferior quadrant appeared to be the highest-ranked feature by the feature selection methods.

### Differential diagnosis

#### Glaucoma staging

The separation of the different stages of glaucoma was performed based on two approaches: one versus rest (OvR) and one versus one (OvO) model training using three different classifiers, namely KNN, SVM and RF using the RNFL, GC-IPL and MC thickness features (see Table 2). In the OvR approach, all stages of glaucoma and normal were used for training, considering four possible two-class classification problems: early versus the rest (moderate, advanced, normal), moderate versus the rest (early, advanced, normal), advanced versus the rest (early, moderate, normal), and normal versus the rest (early, moderate, advanced). The performance of the diagnostic model was evaluated using multiple metrics, i.e., sensitivity, specificity, accuracy, AUC and F1-score. Higher sensitivity with a lower specificity may cause higher false positives and thus result in overdiagnosis; however, AUC best summarises diagnostic performance in terms of both sensitivity and specificity, so we have focused on that for comparative analysis. From the comparative performance of the three classification models (Table 2), it is evident that the RF classifier outperformed the others in terms of AUC, with an AUC of 0.84 (95% CI: 0.78–0.89), 0.88 (95% CI: 0.85–0.91), 0.96 (95% CI: 0.96–0.99) for early, moderate and advanced glaucoma patients (Supplementary Fig. 4(a), Table 2).

**Table 2:** Performance of the three different classification models for diagnosing glaucoma stages with fivefold cross validation.

		One vs rest approach			One vs one approach		
Classifier	Metric	Early vs rest	Moderate vs rest	Advanced vs rest	Early vs Normal	Moderate vs Normal	Advanced vs Normal
KNN	Sensitivity	0.830 ± 0.092	0.848 ± 0.080	**0.920 ± 0.097**	0.855 ± 0.049	0.771 ± 0.094	0.890 ± 0.127
	Specificity	0.657 ± 0.017	0.768 ± 0.020	0.808 ± 0.024	0.879 ± 0.061	**0.985 ± 0.021**	1 ± 0
	Accuracy	0.743 ± 0.055	0.808 ± 0.050	0.864 ± 0.061	0.867 ± 0.037	0.878 ± 0.054	0.945 ± 0.063
	AUC	0.810 ± 0.050	0.837 ± 0.048	0.917 ± 0.068	0.924 ± 0.028	0.880 ± 0.050	0.945 ± 0.063
	F1-Score	0.781 ± 0.067	0.844 ± 0.034	0.908 ± 0.075	0.773 ± 0.067	0.836 ± 0.094	0.938 ± 0.074
SVM	Sensitivity	0.713 ± 0.116	**0.923 ± 0.084**	0.900 ± 0.089	0.907 ± 0.070	**0.959 ± 0.039**	**1 ± 0**
	Specificity	**0.747 ± 0.037**	0.606 ± 0.062	0.838 ± 0.028	**0.920 ± 0.077**	0.969 ± 0.034	**1 ± 0**
	Accuracy	0.730 ± 0.077	0.764 ± 0.073	0.869 ± 0.045	0.913 ± 0.021	0.964 ± 0.028	**1 ± 0**
	AUC	0.801 ± 0.058	0.812 ± 0.061	0.914 ± 0.057	**0.961 ± 0.018**	0.978 ± 0.021	**1 ± 0**
	F1-Score	0.773 ± 0.032	0.807 ± 0.040	0.903 ± 0.052	**0.875 ± 0.038**	**0.947 ± 0.039**	**1 ± 0**
RF	Sensitivity	**0.830 ± 0.083**	0.880 ± 0.104	**0.920 ± 0.098**	**0.909 ± 0.057**	0.936 ± 0.039	**1 ± 0**
	Specificity	0.737 ± 0.021	**0.788 ± 0.030**	**0.869 ± 0.061**	0.920 ± 0.044	0.970 ± 0.021	0.997 ± 0.007
	Accuracy	**0.784 ± 0.052**	**0.834 ± 0.052**	**0.894 ± 0.049**	**0.915 ± 0.020**	**0.953 ± 0.014**	0.998 ± 0.003
	AUC	**0.836 ± 0.054**	**0.882 ± 0.030**	**0.963 ± 0.025**	0.946 ± 0.035	**0.982 ± 0.020**	**1 ± 0**
	F1-Score	**0.798 ± 0.056**	**0.876 ± 0.033**	**0.958 ± 0.016**	0.849 ± 0.042	0.927 ± 0.028	0.990 ± 0.021
AVERAGE	Sensitivity	0.791 ± 0.083	0.884 ± 0.104	0.913 ± 0.098	0.890 ± 0.059	0.889 ± 0.057	0.963 ± 0.042
	Specificity	0.714 ± 0.021	0.721 ± 0.03	0.838 ± 0.061	0.906 ± 0.061	0.975 ± 0.025	0.999 ± 0.002
	Accuracy	0.752 ± 0.052	0.802 ± 0.052	0.876 ± 0.049	0.898 ± 0.026	0.932 ± 0.032	0.981 ± 0.022
	AUC	0.816 ± 0.054	0.844 ± 0.030	0.932 ± 0.025	0.944 ± 0.027	0.947 ± 0.030	0.982 ± 0.021
	F1-Score	0.784 ± 0.056	0.842 ± 0.033	0.923 ± 0.016	0.832 ± 0.049	0.903 ± 0.053	0.976 ± 0.032

Significant values are in bold.

The reported numbers are mean ± standard deviation over the 5 folds. Input features included RNFL, GC-IPL and Macular thickness features. Advanced glaucoma cases included are only based on mean deviation of visual fields. The highlighted numbers represent highest performance (for each metric) among the classifiers. KNN: K-Nearest Neighbours, SVM: Support Vector Machines, RF: Random Forests.

On the other hand, in the OvO approach, three separate binary class problems were considered: early versus normal, moderate versus normal, and advanced versus normal. From the comparative performance of the three classification models (Table 2, and Supplementary Fig. 4(b)), it is evident that SVM and RF outperformed the KNN classifier in terms of AUC. For early glaucoma, we achieved the best AUC of 0.96 (95% CI: 0.95–0.98) using SVM; for moderate glaucoma, an AUC of 0.98 using both RF (95% CI: 0.96–1.00) and SVM (95% CI: 0.96–1.00); and for advanced glaucoma, an AUC of 1.00 (95% CI: 1.00–1.00) using both RF and SVM (Supplementary Fig. 4(b), Table 2). A sub-analysis was performed with advanced glaucoma identified based on CFD and MD of visual fields (Supplementary Fig. 5).

#### Overall glaucoma

Overall, binary classification was performed between whole glaucoma (early, moderate, and advanced) and normal using five-fold cross-validation (Supplementary Table 4, Supplementary Fig. 6). The outcomes indicate that SVM outperformed the other ML models and produced a mean sensitivity of 0.91 (95% CI: 0.87–0.94), specificity of 0.97 (95% CI: 0.95–0.99), accuracy of 94.0% (95% CI: 0.91–0.97) and AUC of 0.97 (95% CI: 0.95–0.99). On five-fold cross-validation, performance was robust across all the folds (Supplementary Table 4, Supplementary Fig. 6). For further verification, the data was shuffled five times (five-times five-fold cross-validation) and the classifier still produced an AUC of 0.97 which confirms the robustness of the model in glaucoma diagnosis across all the folds (Supplementary Fig. 6). The RF model performed close to the SVM with an AUC of 0.97 (95% CI: 0.95–0.99), while the KNN model provided the least performance for the diagnosis of overall glaucoma with an AUC 0.92 (95% CI: 0.90–0.95). A sub-analysis using the original artefact-free data without MICE-based imputation and SMOTE-based oversampling showed no significant difference in performance (see Supplementary Results and corresponding Supplementary Tables 5 and 6).

#### Sub-analysis using RNFL, GC-IPL and MC thickness feature sets

A sub-analysis was performed to compare the subset of selected features obtained from RNFL, GC-IPL and MC thickness analysis (Supplementary Fig. 11). The subset of features consisted of the RNFL feature set (49 features), GC-IPL feature set (8 features) and MC thickness feature set (9 features). Dual and triple combinations of the feature sets were also compared, i.e. RNFL + GC-IPL (57 features), RNFL + MC thickness features (58 features), and all features RNFL + GC-IPL + MC (66 features). Using a RF classifier, the sub-analysis comparing the singular feature sets showed that RNFL performs better (AUC: 0.92, 95% CI: 0.88–0.96) than GC-IPL (AUC: 0.89, 95% CI: 0.88–0.90) and MC thickness (AUC: 0.80, 95% CI: 0.78–0.83) feature sets, with MC thickness being the worst performer (AUC: 0.80). Comparing the dual and triple combination of the feature sets, we observed that the RNFL + GC-IPL combination of feature sets produced similar performance (AUC: 0.97, 95% CI: 0.95–0.99) as the triple combination using MC (RNFL + GC-IPL + MC) (AUC: 0.97, 95% CI: 0.95–0.99), with GC-IPL + MC being the worst performer (AUC: 0.86, 95% CI: 0.84–0.87). Considering this, RNFL + GC-IPL feature sets were used to train the other models and compare the machine performance with the human expert.

#### Comparing human vs machine performance

A sub-analysis was performed to compare human performance with ML (Table 3 and Fig. 2 (b–c)). In this case, only RNFL and GC-IPL data was used, as the inclusion of MC thickness data does not change the machine performance significantly (Supplementary Fig. 11). The performance of the clinicians was evaluated on the analysed image dataset using CIRUS HD OCT (RNFL and GC-IPL analysed data containing both numbers and images) in three ways by three clinicians based at CFEH: first, the deviation maps and colour OCT maps were masked except for the thickness values (numbers) used by the machine (masked, Supplementary Fig. 7(a)). Second, all the relevant information including colour maps and deviation maps were revealed to grade on the images (unmasked-sequential, Supplementary Fig. 7(b)). Finally, one of the clinicians was provided with unmasked data with random distribution again (unmasked-random, Supplementary Fig. 7(c)). The performance metrics were calculated for the clinicians and compared with the machine performance (Table 3 and Supplementary Table 7). It is important to note that CFEH is a referral-only, optometry-ophthalmology service, providing assessment of patients who are suspected of having eye disease of the visual pathways, or those who have a disease including glaucoma. The clinicians are highly trained in ocular assessment and interpreting imaging. The three clinicians who graded the images had 12, 11 and 6 years of experience at CFEH.

**Table 3:** Comparative performance for human (clinicians) vs machine (ML-based models) for diagnosing glaucoma.

Decision maker	Stage	Sensitivity	Specificity	Accuracy	AUC	F1-Score
Machine (RF)	Overall Glaucoma	0.917 ± 0.069	0.958 ± 0.018	0.938 ± 0.033	0.967 ± 0.025	0.921 ± 0.038
	Early	**0.909 ± 0.057**	0.934 ± 0.044	**0.921 ± 0.024**	0.947 ± 0.034	0.860 ± 0.057
	Moderate	**0.959 ± 0.039**	0.960 ± 0.039	0.960 ± 0.025	**0.982 ± 0.017**	0.921 ± 0.035
	Advanced	**1 ± 0**	0.997 ± 0.007	0.998 ± 0.003	**1 ± 0**	0.990 ± 0.021
Machine (SVM)	Overall Glaucoma	**0.919 ± 0.051**	**0.974 ± 0.031**	**0.946 ± 0.023**	**0.972 ± 0.018**	**0.935 ± 0.028**
	Early	0.874 ± 0.052	0.962 ± 0.037	0.918 ± 0.015	**0.956 ± 0.021**	**0.880 ± 0.027**
	Moderate	0.959 ± 0.038	**0.969 ± 0.037**	**0.964 ± 0.031**	0.978 ± 0.020	**0.945 ± 0.044**
	Advanced	**1 ± 0**	**1 ± 0**	**1 ± 0**	**1 ± 0**	**1 ± 0**
Machine (KNN)	Overall Glaucoma	0.808 ± 0.071	0.963 ± 0.029	0.886 ± 0.032	0.926 ± 0.034	0.864 ± 0.038
	Early	0.680 ± 0.115	0.910 ± 0.058	0.795 ± 0.050	0.817 ± 0.051	0.689 ± 0.047
	Moderate	0.828 ± 0.052	0.976 ± 0.016	0.902 ± 0.021	0.908 ± 0.025	0.855 ± 0.025
	Advanced	0.861 ± 0.111	1 ± 0	0.931 ± 0.055	0.931 ± 0.055	0.922 ± 0.065
Clinician-1 (Masked)	Overall Glaucoma	0.797	0.838	0.816	-	0.816
	Early	0.444	0.864	0.774	-	0.457
	Moderate	0.714	**0.966**	**0.917**	-	0.769
	Advanced	**1**	**1**	**1**	-	**1**
Clinician -2 (Masked)	Overall Glaucoma	**0.863**	**0.842**	**0.853**	-	**0.862**
	Early	0.571	**0.893**	**0.816**	-	**0.600**
	Moderate	0.800	0.936	**0.917**	-	0.727
	Advanced	**1**	**1**	**1**	**-**	**1**
Clinician -3 (Masked)	Overall Glaucoma	0.826	**0.842**	0.834	-	0.837
	Early	0.500	**0.893**	0.795	-	0.550
	Moderate	0.777	0.951	0.929	-	**0.786**
	Advanced	**1**	0.983	0.985	-	0.947
Mean Clinician Performance (Masked)	Overall Glaucoma	0.826	0.842	0.834	-	0.838
	Early	0.500	0.893	0.795	-	0.550
	Moderate	0.777	0.951	0.929	-	0.786
	Advanced	1	0.983	0.985	-	0.947

Performance is reported using a one-vs-one approach for clinicians (using RNFL and GC-IPL data). Highlighted numbers were used to compare the overall performance in terms of accuracy. Reported: mean ± standard deviation.

Significant values are bold.

The sensitivity and specificity obtained from the clinicians (Table 3) are plotted in the ROC curve to compare with machine performance (Fig. 2). It shows that with the masked data, the clinicians had a sensitivity ranging from 44.4–57.1% and specificity ranging from 86.4%–89.3% for early glaucoma, sensitivity of 71.4%–80.0% and specificity of 93.6–96.6% for moderate glaucoma, and sensitivity of 100.0% and specificity of 98.3–100.0% for advanced glaucoma. While diagnosing overall glaucoma by the clinicians, they have a sensitivity ranging from 79.7–86.3% and specificity ranging from 83.8–84.2% using the masked data.

Overall, ML outperforms mean human performance while diagnosing early and moderate glaucoma by 12.3% (SVM)–12.6% (RF) and 3.1% (RF)–3.5% (SVM) in terms of accuracy. However, the performance for advanced glaucoma is somewhat similar for both human and machine. For overall glaucoma diagnosis, ML accuracy was 10.4% (RF)–11.2% (SVM) and 9.2% (RF)–10.0% (SVM) higher than the human expert using the masked and sequential unmasked data respectively. For both glaucoma staging and overall glaucoma diagnosis, the clinician’s performance for sequentially unmasked and random unmasked data was somewhat close; however, it was substantially lower than the machine’s performance, especially while diagnosing early glaucoma (Supplementary Fig. 8 & Supplementary Fig. 9).

### Explainable ML results

#### SHAP analysis

**Fig. 3:**
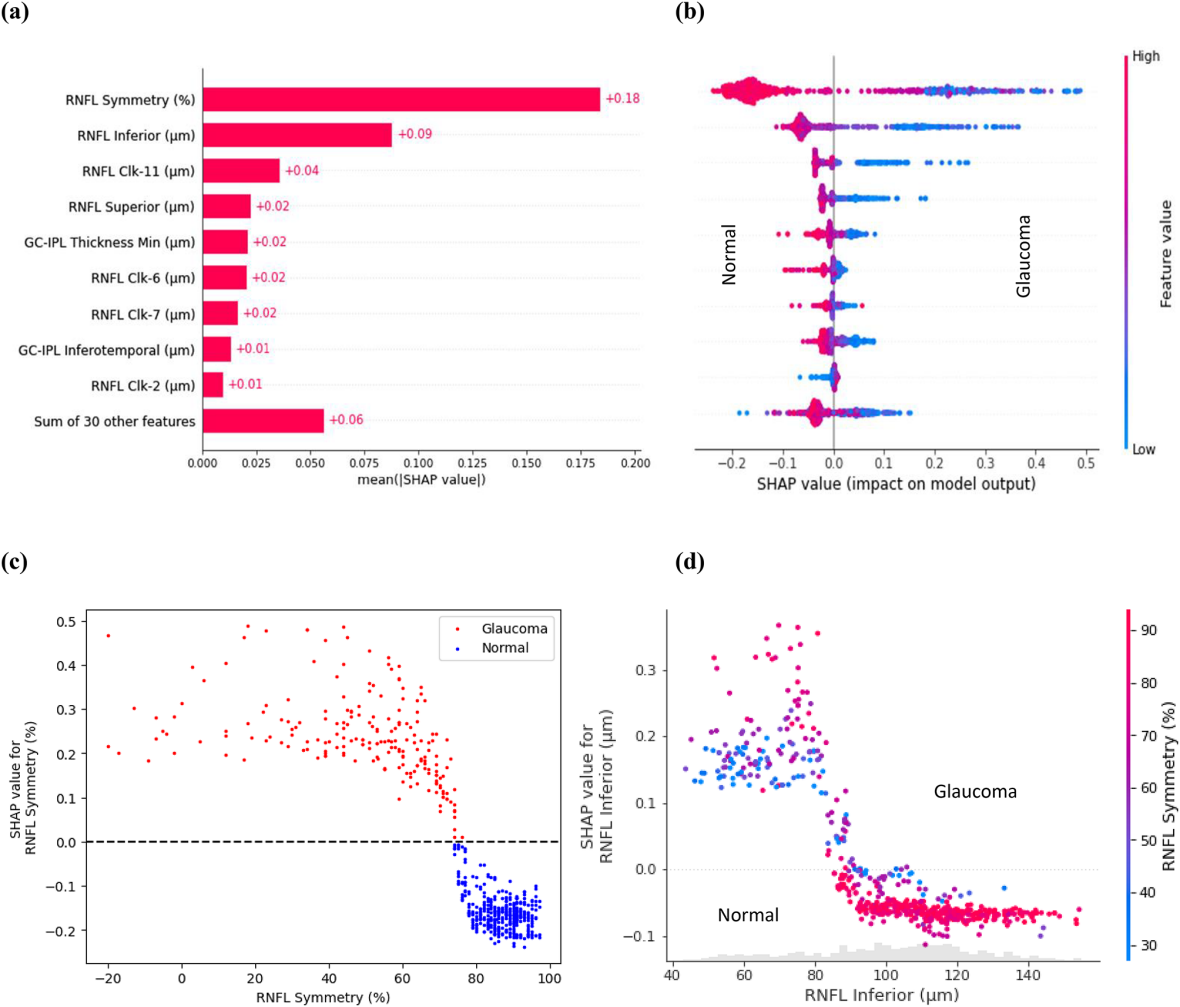
SHAP analysis results. (**a**) SHAP feature ranking and (**b**) SHAP summary plot. The top-ranked features are RNFL symmetry, RNFL inferior, RNFL clock-11 (part of the superior quadrant) and RNFL-superior thickness. The blue colour represents lower magnitudes of feature values while the red colour represents higher values. positive SHAP values on the right side push the model to predict a win (in this case the probability of being classified as glaucoma), while negative SHAP values on the left push the model to predict a loss (probability of being classified as normal). (c) SHAP dependence plot (RNFL symmetry). Positive SHAP values on the vertical axis mean that the corresponding feature values have a positive impact on the prediction, while negative SHAP values mean that the feature values have a negative impact. Points close to the zero line can be useful for identifying instances where a feature did not contribute to the model’s prediction. (**d**) SHAP dependence plot with interaction visualisation (interaction between RNFL inferior and RNFL symmetry). For (**c**), the data points in red above the horizontal dashed line represent the samples classified as glaucoma, while those in blue below the line represents the samples classified as normal. In (**d**), the red points corresponding to higher RNFL inferior thickness indicate the presence of higher RNFL symmetry, which generates negative SHAP values and shows a lower chance of being classified as glaucoma. RNFL clock hours-2,6,7,11 represents RNFL thickness measured in clock-hour sectors. Each sector is measured in degrees, usually in increments of 30 degrees, creating a 360-degree circle around the optic nerve head.

SHAP analysis was performed on the dataset containing the included spatial domain features used to train the RF model. The goal was to generate a global feature rank based on the mean of absolute SHAP values per feature, corresponding to all samples from each class and sorted in descending order (Fig. 3). The SHAP-based global feature ranking (Fig. 3 (a)) and summary plot (Fig. 3 (b)) show that RNFL symmetry, RNFL thickness at the inferior quadrant, RNFL clock-11 (part of RNFL superior), and RNFL superior thickness have a higher impact on the classification. The SHAP summary plot (Fig. 3 (b)) further indicates that the lower the RNFL symmetry, the higher the chances of being classified as glaucoma. Similarly, the lower the RNFL inferior/ RNFL clock-11 /superior thickness, the higher the chances of being classified as glaucoma.

To understand the impact of each feature in the model’s prediction for every data sample, a SHAP dependence plot was generated, illustrating the relationship between the feature’s magnitude and its contribution to the model prediction. The SHAP dependence plot was created for the two top-ranked OCT features, i.e., RNFL symmetry and RNFL inferior thickness (Fig. 3(c, d)). The SHAP dependence plot (Fig. 3 (c)) confirms that lower RNFL symmetry increases the chance of a sample being classified as glaucoma (points corresponding to positive SHAP values on the vertical axis), whereas higher RNFL symmetry increases the chance of being classified as normal (points corresponding to the negative SHAP values), with a crossover point of around 71. While RNFL Symmetry is the highest-impact feature, the SHAP interaction visualisation plot between RNFL inferior thickness and RNFL symmetry further clarifies the interpretation (Fig. 3(d)). In the cases where the RNFL inferior thickness is lower, a lower RNFL symmetry (i.e., higher asymmetry) increases the chances of the patient being diagnosed with glaucoma. Conversely, higher symmetry reduces the chances of the instances being classified as glaucoma.

#### Partial dependency analysis

**Fig. 4:**
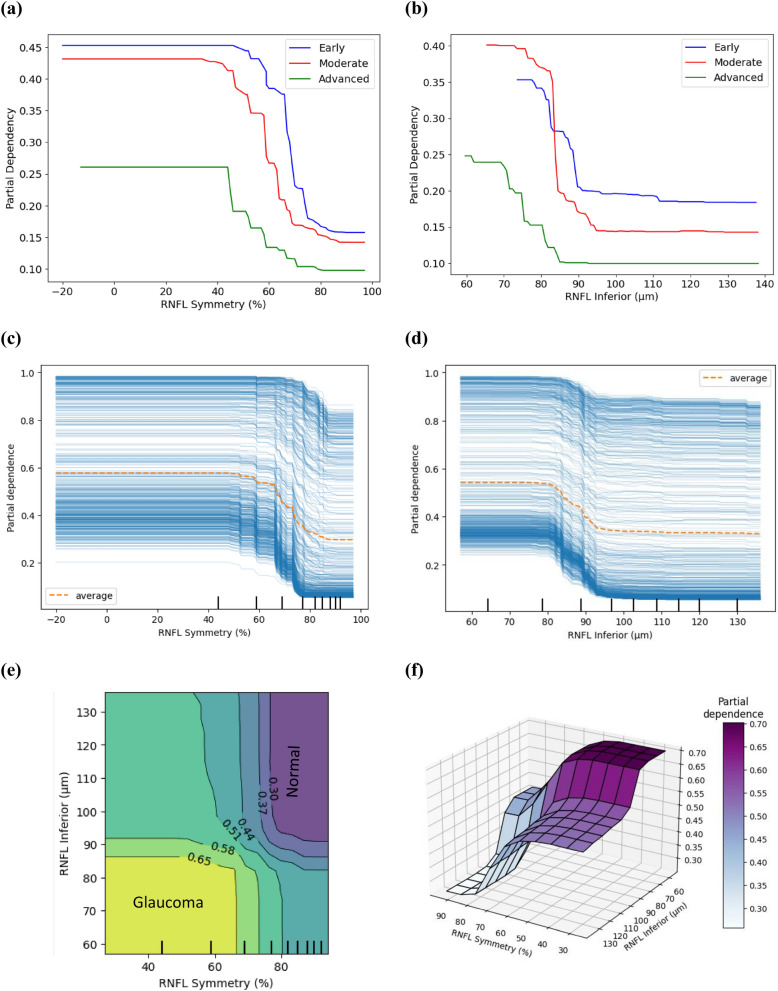
(**a**) Partial dependency plot (PDP) comparison of early, moderate, and advanced glaucoma for RNFL Symmetry (b) PDP for RNFL Inferior (**c**) PDP and individual conditional expectation (ICE) Plots for RNFL symmetry (estimated cut-off = 71%), (d) PDP and ICE plots for RNFL inferior (estimated cut-off = 88.13 μm), (**e**) two-way numerical PDP for RNFL symmetry and RNFL inferior (f) 3D feature interaction plot for RNFL symmetry and RNFL inferior. In all cases (**a**–**f**), the y-axis represents the predicted probability of a machine learning model, and the x-axis presents the magnitude of feature values. For Individual Conditional Expectation (ICE) plots (**b**,**d**), the thin separate curves show the dependency of the prediction on the feature (individual dependence from each sample) and the thick curves represent the average effect (mean partial dependence).

To understand the marginal effect of the feature values on the predicted output of the classification model, both individual component expectation (ICE) and partial dependence plot (PDP) plots were created (Fig. 4 (a–f)). The decision boundaries for early, moderate and advanced glaucoma patients are illustrated in the combined PDPs for early, moderate and advanced glaucoma patients (Fig. 4 (a-b)) for the top features ranked by SHAP analysis: RNFL symmetry and RNFL inferior thickness. The relative comparison shows that the cut-off for the decision exhibits a trend from right to left, i.e., the lower the symmetry and the lower the inferior quadrant thickness, the higher the chances of disease severity.

To better understand the overall dependency, plots were drawn for overall glaucoma diagnosis, considering mean prediction (PDP) and prediction for every sample (ICE). From the ICE and PDP plots (Fig. 4 (c–d)), it can be observed that there is a clear descending slope between 60–80% and 85–90 μm respectively for RNFL symmetry and RNFL inferior thickness feature values, where the higher probability (left) indicates higher chances of being classified as glaucoma and lower probability (right) represents chances of being classified as normal. The comparison of the magnitude of the slope indicates that RNFL inferior thickness (strong slope, Fig. 4d) has a higher contribution towards classification than RNFL superior (moderate slope, Supplementary Fig. 10e). Similarly, GC-IPL thickness at the inferotemporal area has a higher contribution than the supertemporal area (Supplementary Fig. 10g,i). Based on the predicted probability of each feature by PDPs, a horizontal line was drawn at the decision boundary and thus cut-off values were estimated at the intersection point of the decision boundary and the PDPs (Supplementary Fig. 10).

A two-way interaction plot was created to understand the dependency of the predicted outcome on multiple top-ranked features and interaction between themselves, e.g., RNFL symmetry and RNFL inferior (Fig. 4 (e)). From the two-way interaction plot (Fig. 4e), we observe that if the RNFL symmetry is below about 71%, this feature is dependent on RNFL inferior thickness, and their combined contribution creates a higher probability of patients being classified as glaucoma (predicted probability > 0.58). Above RNFL symmetry 71% (vertical contour), the RNFL inferior thickness is independent of RNFL symmetry and there is a higher possibility of patients being classified as normal (predicted probability < 0.51). The 3D feature interaction plots (Fig. 4 (f)) show the combined effect of each of the two features on the prediction outcome, i.e., the dependency of RNFL symmetry on RNFL inferior thickness.

### App development

Using the explainable results, two tools were developed to assist clinicians in glaucoma diagnosis. The first one is an Excel-based tool, utilising SHAP feature importance and PDA-based cut-off and estimated probabilities. The second one is an RF model deployed as a web app, which is trained with SHAP-based most important features and allows the user to input those feature values.

#### Glaucoma likelihood calculator as an excel app

**Fig. 5:**
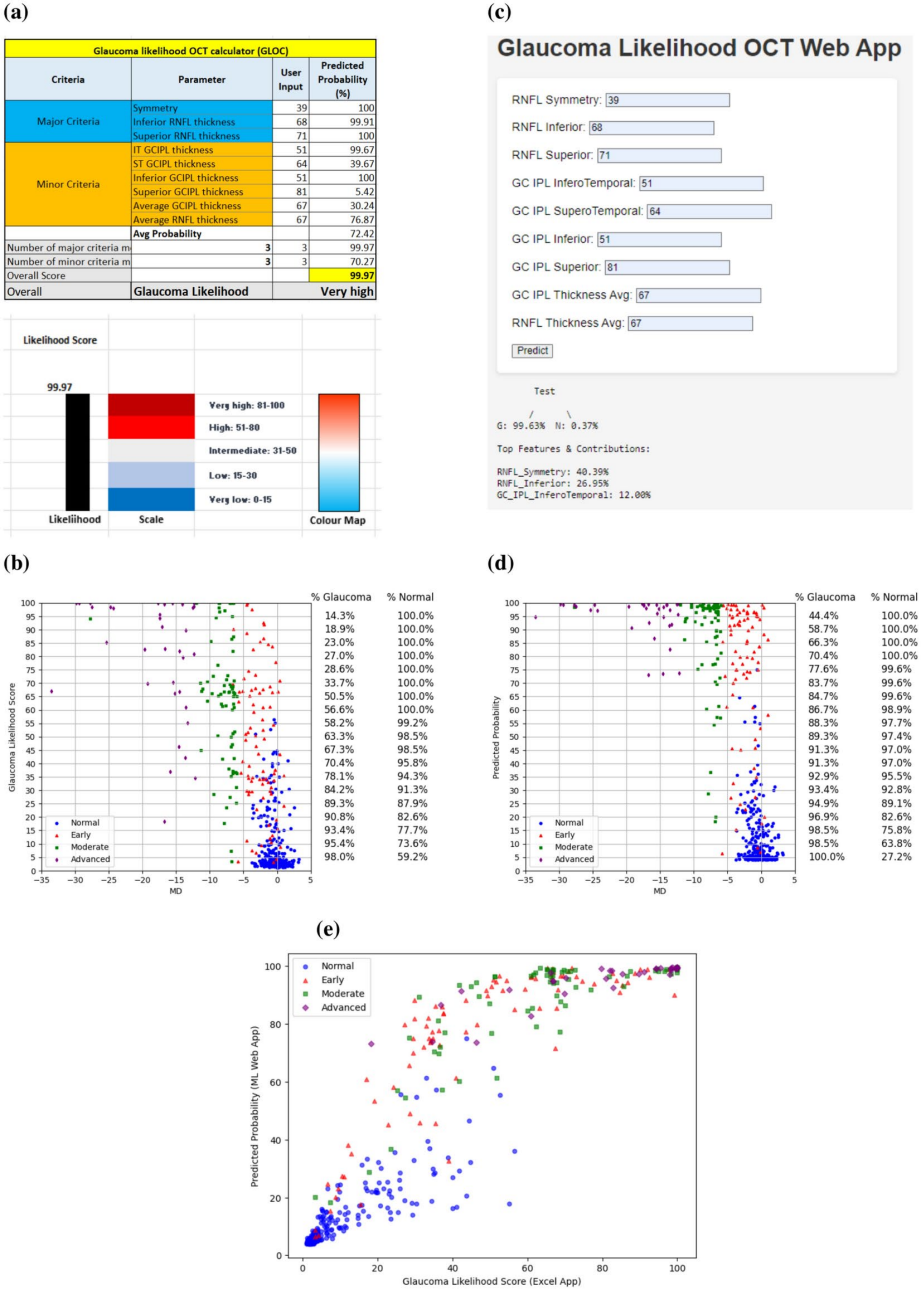
(**a**) Snapshot of the Glaucoma likelihood OCT calculator (GLOC) based on XAI (partial dependency analysis-based predicted probabilities and SHAP-based feature ranking using the RF classifier). (**b**) Plot of glaucoma likelihood scores versus mean deviation of visual fields. The % values on the right indicate the % of glaucoma cases above that threshold, and the % of normal cases below that threshold. (**c**) Snapshot of the Glaucoma likelihood OCT web app based on a RF model deployed on the web. (**d**) Plot of predicted probabilities for glaucoma versus mean deviation of visual fields. The % values on the right indicate the % of glaucoma cases above that threshold and the % of normal cases below that threshold. (**e**) Correlation between glaucoma likelihood score by the XAI-based Excel app and predicted probability by the RF deployed web app. Demographics: 195 glaucoma eyes (early:86, moderate:72, advanced based on MD:37), 261 normal eyes. The other normal subjects’ MD values were unavailable.

To develop the Excel app, the feature ranking provided by the SHAP analysis was categorised into major and minor criteria, based on previous literature and the clinicians’ experience. Major features included those suggested by the SHAP analysis: RNFL symmetry, inferior RNFL thickness and superior RNFL thickness. Minor criteria included the inferotemporal and supertemporal GC-IPL thickness, inferior and superior GC-IPL thickness, and average RNFL and GC-IPL thickness. RNFL clock hours (e.g., RNFL clk-11, RNFL clk-6 & 7) were excluded as they are already part of superior and inferior RNFL thickness. The decision boundary cut-off for the given features were estimated using partial dependency plots (Supplementary Fig. 10). The numerical cut-off and estimated probabilities by PDA corresponding to each feature were provided in the back end, and the Excel program operates based on weighted outcomes on major and minor criteria (Fig. 1 (b)). At the front end, the clinicians can see a live scale (e.g., very low, low, intermediate, high, very high) with the average likelihood of glaucoma in percentage (%) (Fig. 5 (a)). The plot shows that using the likelihood probability score cut-off 0.25 (considering probability > 25% as glaucoma and probability < 25% as normal), the tool generates a sensitivity of 89.3%, specificity of 87.9% and accuracy of 88.5%. This implies that above the 0.25 probability cut-off, the app identifies around 90% of the glaucoma patients as glaucoma and 88% normal patients as normal. Moving the threshold cutoff higher (upwards) causes lower sensitivity and higher specificity; on the other hand, moving the threshold downwards produces a higher sensitivity and lower specificity.

#### Ml-Model deployment as a web app

To validate our proposed XAI-based Excel app, a second, web-based application was developed, where an RF model was deployed using the SHAP-ranked most important features (all major and minor features described above). The key difference of this web-based app from the Excel-based app is the method of prediction of glaucoma likelihood. While the Excel-based app produces a likelihood score based on a weighted outcome of major and minor criteria based on PDA, in the web app we allow the machine learning model to predict the probability based on the same inputs (Fig. 5(d)). The plot shows a better probability distribution classifying ‘glaucoma’ and ‘normals’ at a probability cut-off 0.5 (sensitivity = 89.3%, specificity = 97.4%, accuracy = 90.1%); however, for most of the early and moderate glaucoma samples, the predicted probabilities range from 60–100%.

It is worth mentioning that the web app not only generates predicted probabilities with classification, it produces local explainable results based on SHAP-analysis (for the test sample), which shows the top three features and their percentage of contributions towards the classification. This functionality enables clinicians to revisit and scrutinise specific features (e.g., RNFL Symmetry, RNFL Inferior and GC-IPL Inferotemporal thickness in the example of Fig. 5c, with predicted probabilities in Fig. 5 (d)), which significantly affect the classification or diagnosis. Unlike the SHAP-based global feature importance, this local feature importance may vary for different test patients.

A correlation analysis was performed on the glaucoma likelihood using our proposed Excel-based tool and predicted probabilities produced by the web-based ML-app (Fig. 5e). The correlation analysis produces a correlation coefficient of r = 0.92, which implies a strong positive correlation between our proposed Excel-app and ML-deployed explainable web app and validates its performance with the given data (Fig. 5 (e)). Additionally, a correlation analysis was conducted between the predicted probability scores of the non-explainable (trained with 66 spatial-frequency domain features) and SHAP-based explainable machine learning RF model deployed on the web (trained with the limited 9 spatial domain features). The analysis revealed a correlation coefficient of r = 0.98, indicating a strong correlation between the explainable and non-explainable models.

## Discussion

The present study shows a comparative analysis of glaucoma staging and overall glaucoma diagnosis of human and machines. The results obtained from the study can be discussed from three angles: performance of differential diagnosis, implications of the research findings, and the study limitations and possible future research directions. First, using the same dataset, the analysis shows that machine performance surpasses human performance by 10.4 (RF)–11.2 (SVM)%, 12.3 (SVM)–12.6% (RF) and 3.1 (RF)–3.5 (SVM)% in terms of accuracy when diagnosing overall, early and moderate glaucoma respectively; however, the performance of advanced glaucoma diagnosis shows identical performance for both human and machines. The clinicians faced challenges in distinguishing early glaucoma from normal cases, where subtle differences in retinal features are not visually discernible. This makes detecting early glaucoma particularly critical, yet subjective and prone to variability among clinicians. In contrast, the machine learning model excelled in these cases, demonstrating its potential to assist clinicians by providing consistent and reliable support in decision-making.

Second, the present study outperforms recent ML and DL-based studies using a limited number of features (Table 4). Among the ML-based studies, Wu et al.^21^ used an SVM classifier for glaucoma staging using 114 OCT features (Spectralis OCT) and achieved an AUC of 0.78, 0.89 and 0.93 respectively for early, moderate and advanced glaucoma. Kooner et al.^69^ used 64 OCTA and other clinical features using an XGBoost classifier and achieved an overall accuracy of 71.3% in differentiating early, mild, moderate and advanced glaucoma eyes, and 83.9% accuracy in differentiation in glaucoma versus normal. Similarly, Xu et al.^70^ used 3D circumpapillary RNFL (cpRNFL) thickness (4 quadrants) measurements plus 68 super-pixel-based features and achieved AUCs of 0.71–0.86 differentiating normal subjects from glaucoma subjects and 0.87–0.90 differentiating glaucoma versus normal. They reported no significant difference in differentiating glaucoma versus normal subjects and glaucoma suspects versus normal subjects. Among the DL-based studies, George et al.^71^ used 3D-OCT features extracted using convolutional neural networks (CNNs) and obtained an AUC of 0.87 using a baseline CNN and 0.94 using their proposed 3D CNN using their attention-guided component. Juneja et al.^72^ used 513 features, including Statistical (18), Grey Level Co-occurrence Matrix (GLCM) (23) and Run Length Matrix (GLRM) (16) and wavelet filter features (456)) from 3D OCT and obtained a precision ranging from 0.91–0.95 and sensitivity ranging from 0.91–0.97 differentiating glaucoma versus normal cases. Panda et al.^73^ used raw Spectralis OCT volume scans applied to an autoencoder-based deep model with principal component analysis and obtained a sensitivity of 90% and an accuracy of 92% with a five-fold cross-validation. Conversely, we have used only 66 OCT spatial-frequency domain features and achieved an AUC of 0.97 (95% CI: 0.95–0.99), 0.96 (95% CI: 0.95–0.98) (SVM), 0.98 (95% CI: 0.96–1.00) (both SVM and RF), and 1.00 (95% CI: 1.00–1.00) (both RF and SVM) for differentiating overall, early, moderate and advanced glaucoma from normal subjects, which is higher compared to the aforementioned studies. However, we acknowledge that direct comparisons with existing machine and deep learning-based studies are limited by differences in datasets, input types, and experimental settings. A benchmarking study is using the same dataset and evaluation protocol, would be required for a more definitive assessment of relative performance.

**Table 4:** Comparison of diagnostic performance for glaucoma diagnosis and staging.

Author(s)	Modality & #Features	Dataset (Country)	Instrument	Demographics (Number of patients, Ethnicity, Age, Sex)	ML/DL Method	Performance Metrics
Wu et al.^21^	114 OCT features	Fu-Jen Catholic University Hospital, Taiwan	Spectralis OCT (Heidelberg Engineering GmbH) Imaging)	• Glaucoma: Early eyes: 337, Moderate eyes: 73, Severe eyes: 88, Normal eyes: 254 • Ethnicity: Not reported; Age: Normal (52.50 ± 16.19 (129)), Glaucoma (59.20 ± 13.03 (254)) • Sex: Male: 47 (36.4%) Normal, 125 (49.2%) Glaucoma, Female: 82 (63.6%) Normal, 129 (50.8%) glaucoma	• SVM (One vs One)	• AUC Early: 0.78, Moderate:0.89, Severe: 0.93 (One vs One) • Validation: Internal (ten-fold CV)
Kooner et al.^69^	64 OCTA and other clinical features	University of Texas Southwestern Medical Center (UTSW), USA	OCTA (Optovue AvantiXR AngioVueHD scanner (Optovue®, Freemont, CA, USA).) Feature extraction: Angio Analytics software (Version 2018.0.0.18)	• 735 patients (1371 eyes), 462 Healthy Eyes • 909 Glaucomatous eyes (77 ocular hypertension , 160 mild, 156 moderates, 216 severe) • Ethnicity, Age, Sex: Not reported	• XGBoost • Feature importance done with XGBoost, but feature selection was not done	• Accuracy: 0.84 (Healthy vs Glaucoma) • Accuracy mean OvO (Early/Moderate/Advanced): 0.71 • Validation: Internal (ten-fold CV)
Xu et al.^70^	3D SD-OCT 3D circumpapillary RNFL (cpRNFL) thickness (4 quadrants) measurements plus 68 super-pixel based features	University of Pittsburgh Medical Center (UPMC) Eye Center, USA	OCT (Cirrus HD-OCT; Carl Zeiss Meditec, Inc., Dublin, CA)	• Total 96 subjects (192 eyes) • 44 Healthy eyes, 59 Glaucoma suspects eyes, 89 Glaucomatous eyes • Ethnicity, Age, Sex: Not reported	• LogitBoost adaptive boosting	• AUC: Super pixel analysis: 0.86 (Healthy vs Glaucoma Suspects) • cpRNFL features: 0.71 (Healthy vs Glaucoma Suspects) • Super pixel analysis: 0.90 (Healthy vs Glaucoma) • cpRNFL features: 0.87 (Healthy vs Glaucoma) • Validation: Internal (ten-fold CV)
George et al.^71^	3D OCT CNN extracted features (numbers not reported)	New York University and the University of Pittsburgh, USA	Cirrus SD-OCT scanner	• Classification: 3782 and Regression: 10,370 OCT scans • Dataset 1: Total Patients: 555, Healthy patients: 109, Healthy scans: 427; Glaucoma patients: 446, Glaucoma scans: 3355 • Dataset 2: 1678 individuals (multiple visits) • Ethnicity, Age, Sex: Not reported	• Attention-guided DL model (AG-OCT)	• AUC: 3D CNN (with attention-guided component): 0.94 • Baseline model (without attention-guided component): 0.87 • Validation: Internal (five-fold CV)
Juneja et al.^72^	3D OCT (513 features, including Statistical (18), GLCM (23) and GLRM (16) and wavelet filter features (456))	Public dataset (not available as of December 20,224)	(Cirrus HD-OCT; Carl Zeiss Meditec, Inc., Dublin, CA)	• Total OCT volumes: 1110 • Glaucoma cases: 847, Normal cases: 263 • Ethnicity, Age, Sex: Not reported	• Major Voting Fusion Framework (MV), KNN, RF, 3-D CNN	• Precision: 0.95 (MV), 0.94 (KNN), 0.94 (RF), 0.91 (3D CNN) • Recall: 0.97 (MV), 0.97 (KNN), 0.91 (3D CNN) • Validation: Internal (ten-fold CV)
Panda et al.^73^	Spectralis OCT volume scans	Singapore National Eye Centre (Singapore) and Aravind Eye Hospital (Madurai, India)	Spectralis OCT; Heidelberg Engineering, Heidelberg, Germany	• Total: 3,782 subjects (Glaucoma: 2,233, Non-glaucoma:1,549) • Ethnicity: Chinese and Indian; Age: Singapore cohort-1: 60.5 ± 8.5, cohort-2: 59.6 ± 9.9; Indian cohort: 56.8 ± 11.8 • % Male: 49% (Singapore cohort-1), 51% (Singapore cohort-2), 75% (Indian cohort)	• Autoencoder-based segmentation and Principal Component Analysis (Uniform Manifold Approximation and Projection (UMAP))	Glaucoma vs Non-glaucoma • Accuracy: 92.0 ± 2.3% • Sensitivity: 90.0 ± 2.4% (at 95% specificity) • Validation: Internal (five-fold CV)
Hasan et al. (current study)	66 OCT spatial-frequency domain features	Centre for Eye Health (CFEH), University of New South Wales (UNSW), Sydney, Australia	OCT (Cirrus HD-OCT; Carl Zeiss Meditec, Inc., Dublin, CA) Feature extraction tools: CIRRUS HD OCT (spatial domain features) and custom python program (frequency domain features)	• 334 Normal eyes (167 patients) • 268 Glaucomatous eyes (268 patients) • Ethnicity: Mixed (multicultural) • Age: Normal (67.77 ± 6.85), Glaucoma (64.48 ± 11.44) • Sex: Normal (Male: 168 eyes, Female: 166 eyes), Glaucoma (Male: 141 eyes, Female: 127 eyes)	• KNN • SVM • RF	• Sensitivity/Recall: Early: 0.91 (RF), Moderate: 0.97 (SVM), Advanced: 1.00 (SVM); Overall glaucoma: 0.92 (RF) • Specificity: Early: 0.92 (SVM), Moderate: 0.99 (KNN), Advanced: 1.00 (SVM), Overall glaucoma: 0.98 (SVM) • Accuracy: Early 0.92 (RF), Moderate: 0.96 (SVM), Advanced: 1.00 (RF & SVM); Overall glaucoma: 0.94 (SVM) • AUCs: Early: 0.96 (SVM), Moderate: 0.978 (SVM), 0.98 (RF), Advanced: 1.00 (both RF & SVM) (OvO); Overall glaucoma: 0.97 (SVM) • Validation: Internal (five-fold CV)

*ML* Machine Learning, *DL* Deep Learning, *CNN* Convolutional Neural Networks, *KNN* K-Nearest Neighbours, *SVM* Support Vector Machines, *RF* Random Forests.

Third, our study also outperformed clinicians’ performance in glaucoma staging and overall glaucoma diagnosis across multiple trials of clinicians (see clinician’s variability, Supplementary Table 8). For example, in terms of glaucoma staging, Reus et al.^11^ reported that a group of ophthalmologists achieved a sensitivity of 61.9%, 66.2% and 96.8% and a specificity of 95.1% in discriminating early, moderate and severe glaucoma patients respectively. Conversely, we achieved a sensitivity of 90.9%, 93.6% and 100% and a specificity of 92.0%, 97.0% and 99.8% using OCT data, and discriminated early, moderate and severe glaucoma patients using an RF classifier.

The present study varies from other ML-based studies on glaucoma diagnosis in terms of both feature engineering and glaucoma likelihood prediction. First, this study utilised frequency-domain features, transferring the spatial domain TSNIT pattern (local measures) using a fast Fourier transform. The comparison of trained models using RNFL, GC-IPL and MC thickness features sets shows that RNFL outperforms the other, which contains the spatial as well as TSNIT-derived frequency domain features. As such, the frequency domain features such as power spectral density and slope of the harmonics of power spectral density show promise in the diagnosis, which represents the utility of TSNIT data. Second, for glaucoma likelihood prediction, we leveraged the SHAP feature ranking (global explanation) to identify major and minor criteria and predicted probability scores by PDA to decide on glaucoma likelihood and developed an Excel-based app. Last, the local feature importance (local explanations) at the inference phase by SHAP analysis enables clinicians to revisit and scrutinise specific features, which significantly affect the classification or diagnosis. It instils confidence and trust in clinicians regarding the AI-generated results and reduces the time and effort required to review the whole OCT data, which contains hundreds of feature values. We found it was useful for them to look at the specific area of the TSNIT pattern (e.g., RNFL inferior thickness) to quickly identify the changes due to glaucoma. The clinicians gave more attention to the RNFL symmetry being the most crucial feature as flagged by the machine. In the past, clinicians used to give less priority to this information.

However, several limitations should be considered while interpreting the results. First, the model is limited to the CIRRUS and not generally applicable to all OCT machines. Second, the glaucoma cohort in this study was small (imbalance ratio: 0.802). However, SMOTE-based oversampling^53^ was performed to overcome this issue. Additionally, some of the patients had artefacts in CIRRUS RNFL analysis, while GC-IPL and macular thickness data were available and vice-versa. Instead of removing a patient’s whole data when one parameter is artefact-free (e.g., RNFL) and another parameter has an artefact (e.g., GC-IPL), we utilised the MICE^38^ algorithm to impute missing values (3.65% of total samples). The performance metrics with and without the augmented data showed a non-significant difference, which suggests that in our case the original data was already diverse enough and use of imputation and oversampling was not necessary to improve the model’s performance. As imputation and oversampling add computational complexity to the ML pipeline, it may be omitted for a reasonably well-balanced dataset. Also, although imputation and oversampling have been used widely in medical machine learning, a recent study has raised concerns about the clinical validity of using SMOTE in medical data, highlighting the need for further research into advanced modelling techniques^74^. Using a large dataset and exploring alternative imputation techniques such as KNN imputation^75^ and oversampling techniques such as Adaptive Synthetic Sampling (ADASYN)^76^ could be considered as possible future work.

Second, regarding the interpretation techniques used, both SHAP and PDA are computationally demanding. Also, feature independence^62^ is a limitation of PDA with a single feature as observation. However, SHAP addresses this limitation by using a game theoretical approach and considering the feature interaction of multiple high-ranked features. Therefore, being a mixed approach and taking advantage of the decision on multiple major and minor criteria, our proposed multi-feature diagnostic tool overcomes the limitations. Minor variations in the top-ranked features (e.g., RNFL symmetry) are unlikely to significantly affect the model’s classification outcome for several reasons. First, in our Excel-based explainable tool (Fig. 1b and Fig. 5a), decisions are based on a weighted scoring system that combines major and minor criteria. This approach inherently reduces the sensitivity to small variations in any single feature, including RNFL symmetry, by aggregating the contributions of multiple features. Second, the web-based app employs an RF classifier using SHAP-ranked features. RF models, by design, aggregate predictions from multiple decision trees, each of which evaluates features hierarchically based on metrics like the Gini index^77^. This structure ensures that the model’s decisions are robust to minor fluctuations in individual features, such as RNFL symmetry. Last, the web app provides local explanations for individual predictions using SHAP, highlighting the contribution of each feature during the inference/test phase. This transparency allows clinicians to examine specific features, such as RNFL symmetry, and identify any potential biases.

There is an inherent trade-off between explainability and model performance, and achieving a balance is crucial^78^. Using 66 spatial-domain features with an RF-based classifier, we achieved an accuracy of 93.2%. However, when limited to 9 explainable features, the accuracy was 88.2% with the Excel tool and 90.1% with the web app—a difference of 3.1–5%. Despite this slight reduction in performance, our explainable tools successfully balance accuracy with interpretability. By providing both global and local explanations, they enhance trust, reliability, and user-friendliness, making them highly practical for real-world glaucoma diagnosis. However, our focus on ML explainability was limited to using model-agnostic explainable methods (i.e., SHAP^59^ and PDA^64^). It is important to acknowledge other explainable approaches such as Local Interpretable Model-Agnostic Explanations (LIME)^79^. Future research could focus on developing model-specific explainable methods for more robust explanations^80^.

Third, we acknowledge that current versions of the explainable applications are limited to glaucoma diagnosis only (i.e., glaucoma vs normal subjects). The primary reason for excluding glaucoma staging from the app is that the SHAP-based diagnostic approach and PDA are inherently suited for binary classification tasks (e.g., distinguishing between glaucoma and non-glaucoma). Extending these methods to multiclass problems like glaucoma staging introduces significant challenges. SHAP requires separate value computations for each class independently, leading to higher computational complexity and reduced interpretability when combining insights across multiple classes^63,81^. The current implementation of our SHAP-based model focuses on binary classification, which allows a straightforward identification of features critical for diagnosing glaucoma versus non-glaucoma cases. Extending this approach to glaucoma staging would require developing and validating separate SHAP analyses for each stage, which was beyond the scope of this study. Conversely, PDA visualises the effect of each feature on the predicted probability, making it well-suited for binary outcomes where a clear decision boundary can be defined^64,82^. For multiclass staging, PDA would need to account for the interaction of features with multiple decision boundaries, which complicates the interpretation and reduces clinical usability. Future iterations of the app could explore integrating staging capabilities, potentially using advanced methods using hierarchical classification frameworks to design the explainable model for multiclass tasks.

There is scope to extend this work to make it even more useful for future clinical practice. First, the proposed tool simply uses CIRRUS OCT-based RNFL and GC-IPL thickness analysis, which can be used offline for possible interpretation of major and minor important features in glaucoma diagnosis. Future work may include integration of the web-based app with the CIRRUS software or using it as an external application to suggest a predicted probability based on XAI. Second, this study did not consider some important OCT based features, such as cup-to-disc ratio and rim thickness (because CIRRUS does not offer precise measurement); clinical features such as age, gender, medications and intraocular pressure (IOP); or visual field features such as PSD and MD as input features, though some recent studies^83,84^ have shown the utility of these features in diagnosis. Future work may involve fusing these clinical features as functional measures with the structural OCT features. Third, the machine learning models developed in this study do not account for glaucoma suspects and non-glaucomatous optic nerve disease. Future studies should be performed to differentiate glaucoma suspects from glaucoma and normal. For robust detection, multimodal fusion can be performed with colour fundus images and the existing OCT features, which may further improve diagnostic accuracy. In conclusion, the XAI-based developed software tools demonstrated in this study show promise in assisting clinicians and improving differential glaucoma diagnosis with extra reliability.

## Supplementary Information

Supplementary Information.

## Data Availability

The patients provided approval for data to be used as part of our study (UNSW human research ethics approval number: HC210563). Consequently, the raw data are not available for public access, but the statistically analysed OCT data in tabular format for a group of patient cohorts (normal, glaucoma/glaucoma stages) may be accessible upon reasonable request to the corresponding author.
