# Supplementary material for: OCT-based diagnosis of glaucoma and glaucoma stages using explainable machine learning
Source: Sci Rep. 2025 Jan 28;15:3592. doi: 10.1038/s41598-025-87219-w (PMC11775169; doi:10.1038/s41598-025-87219-w)
Supplement: Supplementary file 1 — Supplementary Information. [file 41598_2025_87219_MOESM1_ESM.pdf]

# Supplementary Information

## OCT-based diagnosis of glaucoma and glaucoma stages using explainable machine learning

Md Mahmudul Hasan[a,*], Jack Phu[b,c,d,e], Henrietta Wang[b,c,e], Arcot Sowmya[a], Michael Kalloniatis[b,e,f], Erik Meijering[a]

[a] School of Computer Science and Engineering, University of New South Wales, Sydney, NSW 2052, Australia
[b] School of Optometry and Vision Science, University of New South Wales, Sydney, NSW 2052, Australia
[c] Centre for Eye Health, University of New South Wales, Sydney, NSW 2052, Australia
[d] Faculty of Medicine and Health, University of Sydney, Camperdown, NSW, Australia
[e] School of Medicine (Optometry), Deakin University, Waurn Ponds, VIC, Australia
[f] University of Houston College of Optometry, University of Houston, Houston, TX, United States of America

[*] *Corresponding author*
Md Mahmudul Hasan
School of Computer Science and Engineering
University of New South Wales, Sydney, NSW 2052, Australia
md_mahmudul.hasan@unsw.edu.au

*Short title:* Diagnosis of glaucoma and glaucoma stages using explainable machine learning

**Keywords**: optical coherence tomography, glaucoma, perimetry, explainable machine learning, SHAP analysis, partial dependency analysis

# Supplementary Methods

## Performance Evaluation

### *Performance Measures*

The performance of the machine learning classifiers was evaluated based on their sensitivity or true positive rate (TPR), specificity or true negative rate (TNR), accuracy and F1-score (Equations S1-S4). These parameters were derived from the confusion matrix, which consists of true positive (TP), true negative (TN), false positive (FP) and false negative (FN) values. In the case of diagnosing glaucoma, TP refers to the number of cases that are correctly identified as having glaucoma, while FP refers to the number of cases that are mistakenly identified as having glaucoma. On the other hand, TN indicates the number of cases that are correctly identified as not having glaucoma, while FN represents the number of cases that are incorrectly identified as not having glaucoma when they do. To avoid underfitting due to imbalanced data, the threshold moving approach was used to tune the threshold based on geometric means (Equation S5) to find the optimal point in the sensitivity-specificity (ROC) plot. The sensitivity, specificity, AUC, F1-Score and accuracy score were reported for the optimal point.

$$\text{Sensitivity or TPR or Recall} = \frac{\text{TP}}{\text{TP+FN}} \times 100\% \qquad \text{(S1)}$$

$$\text{Specificity or TNR} = \frac{\text{TN}}{\text{TN+FP}} \times 100\% \qquad \text{(S2)}$$

$$\text{Accuracy} = \frac{\text{TP+ TN}}{\text{TP+TN+FP+FN}} \times 100\% \qquad \text{(S3)}$$

$$\text{F1-Score} = \frac{2 \times \text{Precision} \times \text{Recall}}{\text{Precision+ Recall}} \times 100\% \qquad \text{(S4)}$$

$$\text{Geometric mean} = \sqrt{\text{Sensitivity} \times \text{Specificity}} \qquad \text{(S5)}$$

### *Patient-Level Splitting and Cross-Validation*

To avoid obtaining overly optimistic results, it is generally recommended to not train and evaluate a model's performance on the same data[1,2]. Moreover, the dataset in this study consists of the left and right eye as separate samples for the same patient in some cases. The inclusion of one eye of a specific patient in the training set and the other one of the same patient in the test set may cause data leakage. To avoid this occurring, we adopted patient-level splitting (both eyes of any patient were included either in the training set or in the test set), and five-fold

cross-validation[3] was used to train and test all classification models, of which we report the mean and standard deviations.

# Supplementary Results

**Differential Diagnosis- Advanced Glaucoma**

A sub-analysis was performed with advanced glaucoma identified based on CFD and MD of visual fields using the three different classifiers: KNN, SVM and RF (Supplementary Figure 5). Before deploying ML classifiers on the OCT data, a Mann-Whitney test was performed on the MD values of the two groups, giving a p-value of 0.742, suggesting that the difference between the MD values of the two groups is not significant. The classification results in two ROC plots for two types of sub-classification: advanced glaucoma based on CFD vs normal (Supplementary Figure 5(a)) and advanced glaucoma based on MD vs CFD (Supplementary Figure 5(b)). Using the SVM classifier, we obtained the best AUC of 0.99 (95% CI: 0.99–1.00) using SVM in differentiating advanced glaucoma (based on CFD) from Normal, and 0.793 (95% CI: 0.73–0.85) using RF in differentiating advanced glaucoma (based on MD) from advanced glaucoma (based on CFD).

**Sub-Analysis Using Artefact-Free Original Data**

A sub-analysis was performed using the original artefact free data without MICE-based imputation and SMOTE-based oversampling. All three classifiers were trained and tested with 5-fold cross-validation. The results obtained using the original data were not significantly different than the results obtained using the MICE and SMOTE-augmented data. From the comparative performance of the three classification models' performance on the original data (Supplementary Table 5) using OvR approach, RF outperformed the KNN and SVM classifier in terms of AUC, with mean AUCs of 0.85 (95% CI: 0.80–0.90), 0.87 (95% CI: 0.78–0.96) and 0.95 (95% CI: 0.91–0.99) for early, moderate and advanced glaucoma from the rest with five-fold cross-validation. Using the OvO approach, we achieved a mean AUC of 0.95 (95% CI: 0.89–1.00) (SVM) for early glaucoma, 0.99 (95% CI: 0.95–1.00) (RF) for moderate glaucoma and 1.00 (95% CI:1.00–1.00) (SVM & RF) for advanced glaucoma. For the diagnosis of overall glaucoma versus normal, we achieved the highest sensitivity of 0.92 (95% CI: 0.87–0.97) (RF), specificity if 0.97 (95% CI: 0.95–0.99) (SVM), accuracy of 0.94 (95% CI: 0.92–0.96) (SVM), F1-score of 0.93 (95% CI: 0.91–0.95) (SVM) and an AUC of 0.97 (95% CI: 0.95–0.99) (RF) (Supplementary Table 6). A two-tailed paired t-test comparing the results obtained using the original data and the results obtained using synthetic data shows a non-significant difference ($p>0.05$) across the performance metrics.

# Supplementary Tables

**Supplementary Table 1:** List of features extracted in spatial and frequency domains.

| RNFL thickness Analysis | Count | GC-IPL thickness Analysis | Count | Macular thickness Analysis | Count |
|---|---|---|---|---|---|
| RNFL symmetry (%) | 1 | Average GC-IPL thickness | 1 | ILM-RPE thickness-central subfield | 1 |
| Average RNFL thickness (µm) | 1 | Minimum GC-IPL thickness (µm) | 1 | ILM-RPE thickness-volumetric cube | 1 |
| RNFL thickness ring area (mean) (µm) | 1 | GC-IPL superior (µm) | 1 | ILM-RPE thickness-average cube | 1 |
| RNFL thickness in quadrants (µm) | | GC-IPL superonasal (µm) | 1 | ILM-RPE centre-foveal | 1 |
| • RNFL superior (mean, med, std, skew & kurt) | 5 | GC-IPL inferonasal (µm) | 1 | ILM-RPE inner superior (iSup) | 1 |
| • RNFL nasal (mean, med, std, skew & kurt) | 5 | GC-IPL inferior (µm) | 1 | ILM-RPE inner nasal (iNas) | 1 |
| • RNFL inferior (mean, med, std, skew & kurt) | 5 | GC-IPL inferotemporal (µm) | 1 | ILM-RPE inner inferior (iInf) | 1 |
| • RNFL temporal (mean, med, std, skew & kurt) | 5 | GC-IPL-superotemporal (µm) | 1 | ILM-RPE inner temporal (iTem) | 1 |
| RNFL clock hours | | | | ILM-RPE outer superior (oSup) | 1 |
| RNFL clk 1- 12 | 12 | | | ILM-RPE outer nasal (oNas) | 1 |
| RNFL 256 data points (0 to 256) | | | | ILM-RPE outer inferior (oInf) | 1 |
| • Median | 1 | | | ILM-RPE outer temporal (oTem) | 1 |
| • Standard deviation | 1 | | | | |
| • Skewness | 1 | | | | |
| • Kurtosis | 1 | | | | |
| Rim area | 1 | | | | |
| Disc area | 1 | | | | |
| Average cup to disc ratio | 1 | | | | |
| Vertical cup to disc ratio | 1 | | | | |
| Cup volume | 1 | | | | |
| TSNIT Shannon entropy (information theory) | 1 | | | | |
| TSNIT Fisher information (information theory) | 1 | | | | |
| TSNIT Signal to noise ratio (SNR) (information theory) | 1 | | | | |
| FFT absolute value (real + imaginary) | 1 | | | | |
| FFT real value | 1 | | | | |
| FFT imaginary value | 1 | | | | |
| PSD whole TSNIT (mean, maximum, median, standard deviation, skewness & kurtosis) | | | | | |
| FFT Method | 6 | | | | |
| Welch Method | 6 | | | | |
| Slope of PSD of TSNIT in (1st- 4th) harmonics | | | | | |
| • FFT method | 4 | | | | |

| RNFL thickness Analysis | Count | GC-IPL thickness Analysis | Count | Macular thickness Analysis | Count |
|---|---|---|---|---|---|
| • Welch method | 4 | | | | |
| Slope of PSD of TSNIT (mean, maximum and minimum) (FFT & Welch) | 6 | | | | |
| PSD of RNFL superior quadrant (mean & maximum) (FFT & Welch) | 4 | | | | |
| PSD of RNFL temporal quadrant (mean & max) (FFT & Welch) | 4 | | | | |
| PSD of RNFL nasal quadrant (mean & maximum) (FFT & Welch) | 4 | | | | |
| PSD of RNFL inferior quadrant (mean & max) (FFT & Welch) | 4 | | | | |
| PSD of TSNIT with level-(1~4) DWT (mean & max) | | | | | |
| • FFT method | 8 | | | | |
| • Welch method | 8 | | | | |
| Spectral entropy of PSD of TSNIT (information theory) | 2 | | | | |
| Count (individual analysis) | 111 | | 8 | | 12 |
| Total Count | 131 | | | | |

**Note:** RNFL: Retinal nerve fibre layer, GC-IPL: ganglion cell–inner plexiform layer, ILM-RPE: Inner limiting membrane-retinal pigment epithelium**,** TSNIT: temporal-superior-nasal-inferior-temporal, Med: median, std: standard deviation, skew: skewness, kurt: kurtosis, FFT: Fast Fourier Transform, DWT: Discrete Wavelet transform, PSD: power spectral density ('Welch' and FFT method), max: maximum value, DWT: discrete wavelet transforms ('Haar' wavelet), spatial domain features are above horizontal line, below the line are frequency domain features.

**Supplementary Table 2:** Parameters considered for hyper-parameter tuning for the different classifiers.

| Classifier | Parameter |
|---|---|
| KNN | ▪ Number of neighbours (k) [1–40] |
| SVM | ▪ 'C' value (0.1, 1, 10, 100, 1000) |
| | ▪ Gamma (with 'RBF' kernel) (1, 0.1, 0.01, 0.001, 0.0001) |
| RF | ▪ Number of trees in the forest (n_estimate) [50, 100, 200, 300, 400] |
| | ▪ The maximum depth of the tree (max_depth) [2, 3, 4, 5, 6] |

**Supplementary Table 3**: Features selected from the spatial and frequency domains.

| Feature rank | Spatial domain features | F-value | Feature rank | Frequency domain features | F-value |
|---|---|---|---|---|---|
| 1 | **RNFL symmetry (percentage)** | 502 | 1 | **PSD slope-1 (FFT)** | 292.5 |
| 2 | **RNFL inferior (mean)** | 470.3 | 2 | **FFT absolute** | 275.3 |
| 3 | **RNFL inferior (median)** | 433.1 | 3 | **TSNIT PSD mean (Welch)** | 186.2 |
| 4 | *GC-IPL inferotemporal* | 418.2 | 4 | **TSNIT PSD max (Welch)** | 163.6 |
| 5 | *GC-IPL thickness (minimum)* | 400.1 | 5 | **PSD slope-2 (Welch)** | 138.5 |
| 6 | **RNFL clock 6** | 381.8 | 6 | **Spectral entropy PSD (Welch)** | 132.7 |
| 7 | **RNFL clock 7** | 355.6 | 7 | **PSD maximum slope (Welch)** | 131.8 |
| 8 | **TSNIT (std)** | 343.1 | 8 | **PSD minimum slope (Welch)** | 128.7 |
| 9 | **RNFL thickness (average)** | 315.4 | 9 | **PSD slope-2 (FFT)** | 124.8 |
| 10 | *GC-IPL superior* | 307.5 | 10 | **PSD slope-3 (FFT)** | 118.5 |
| 11 | **TSNIT kurtosis** | 272.8 | 11 | **PSD skewness (FFT)** | 99.7 |
| 12 | *CG-IPL thickness (average*) | 258.9 | 12 | **TSNIT inferior maximum PSD (Welch)** | 99.4 |
| 13 | ILM-RPE outer inferior (oInf) | 241.8 | 13 | **PSD slope-1 (Welch)** | 89 |
| 14 | **TSNIT median** | 235.2 | 14 | **TSNIT inferior mean PSD (Welch)** | 85.5 |
| 15 | **RNFL superior (mean)** | 219.3 | 15 | **Spectral entropy PSD (FFT)** | 68.9 |
| 16 | **RNFL inferior (std)** | 198.8 | 16 | **PSD slope-0 (Welch)** | 56.1 |
| 17 | *GC-IPL superotemporal* | 194.9 | 17 | **TSNIT superior maximum PSD (Welch)** | 43.6 |
| 18 | **RNFL superior (median)** | 188 | 18 | **PSD skewness (Welch)** | 42.7 |
| 19 | **RNFL clock 11** | 171.1 | 19 | **TSNIT superior mean PSD (Welch)** | 40.4 |
| 20 | ILM-RPE thickness Avg Cube | 170.9 | 20 | **TSNIT nasal maximum PSD (Welch)** | 36.4 |
| 21 | *GC-IPL superior* | 153.2 | 21 | **PSD slope-3 (Welch)** | 32.4 |
| 22 | **SNR (dB)** | 140.2 | | | |
| 23 | **RNFL clock 12** | 135.9 | | | |
| 24 | *GC-IPL inferoonasal* | 133.1 | | | |
| 25 | ILM-RPE outer temporal (oTem) | 128 | | | |
| 26 | **RNFL clock 5** | 120.2 | | | |
| 27 | ILM-RPE inner temporal (iTem) | 103.5 | | | |
| 28 | ILM-RPE inner inferior (iInf) | 103 | | | |
| 29 | ILM-RPE outer superior (oSup) | 95.2 | | | |
| 30 | **RNFL thickness (ring scan)** | 89.7 | | | |
| 31 | *GC-IPL superonasal* | 83.6 | | | |
| 32 | **RNFL clock 1** | 73.1 | | | |
| 33 | **RNFL clock 2** | 71.5 | | | |
| 34 | **RNFL nasal (std)** | 63.8 | | | |
| 35 | **RNFL temporal (kurtosis)** | 62.8 | | | |
| 36 | ILM-RPE outer nasal (oNas) | 56.3 | | | |
| 37 | **RNFL nasal (kurtosis)** | 55.6 | | | |
| 38 | **RNFL nasal (skewness)** | 50.1 | | | |

| Feature rank | Spatial domain features | F-value | Feature rank | Frequency domain features | F-value |
|---|---|---|---|---|---|
| 39 | **RNFL temporal (skewness)** | 47.2 | | | |
| 40 | ILM-RPE inner superior (iSup) | 46.5 | | | |
| 41 | **RNFL superior (std)** | 43 | | | |
| 42 | **TSNIT (skewness)** | 40.1 | | | |
| 43 | **RNFL inferior (kurtosis)** | 37.4 | | | |
| 44 | ILM-RPE inner nasal (iNas) | 35.5 | | | |
| 45 | **RNFL nasal (mean)** | 35.1 | | | |

**Note: Boldfaced** features represent RNFL features, *italicised* features indicate GC-IPL features, and features in normal font correspond to MC thickness features**.** RNFL: Retinal nerve fibre layer, GC-IPL: ganglion cell–inner plexiform layer, MC: macular, ILM-RPE: Inner limiting membrane-retinal pigment epithelium**,** TSNIT: temporal-superior-nasal-inferior-temporal; Med: median, std: standard deviation, FFT: Fast Fourier Transform, DWT: Discrete Wavelet transform, PSD: power spectral density ('Welch' and FFT method), DWT: discrete wavelet transforms ('Haar' wavelet)

**Supplementary Table 4:** Performance of the different classifiers in distinguishing overall glaucoma patients versus normal in the different folds of the five-fold cross-validation experiment.

| Approach | Fold | Sensitivity | Specificity | Accuracy | AUC | F1-Score |
|---|---|---|---|---|---|---|
| KNN | Fold-1 | 0.750 | 0.984 | 0.867 | 0.934 | 0.846 |
| | Fold-2 | 0.886 | 0.984 | 0.935 | 0.972 | 0.929 |
| | Fold-3 | 0.763 | 0.897 | 0.830 | 0.888 | 0.813 |
| | Fold-4 | 0.789 | 0.941 | 0.865 | 0.900 | 0.836 |
| | Fold-5 | 0.848 | 0.932 | 0.890 | 0.924 | 0.852 |
| | **Mean ± std** | 0.807 ± 0.058 | 0.948 ± 0.037 | 0.878 ± 0.039 | 0.924 ± 0.033 | 0.855 ± 0.044 |
| | **95% CI** | 0.76–0.86 | 0.92–0.98 | 0.84–0.91 | 0.90–0.95 | 0.82–0.89 |
| SVM | Fold-1 | 0.932 | 0.952 | 0.942 | 0.978 | 0.932 |
| | Fold-2 | 0.955 | 1 | 0.977 | 0.990 | 0.977 |
| | Fold-3 | 0.842 | 0.941 | 0.892 | 0.938 | 0.870 |
| | Fold-4 | 0.895 | 0.985 | 0.940 | 0.952 | 0.932 |
| | Fold-5 | 0.909 | 0.986 | 0.948 | 0.986 | 0.937 |
| | **Mean ± std** | 0.906 ± 0.043 | **0.973 ± 0.025** | **0.940 ± 0.031** | **0.969 ± 0.023** | **0.929 ± 0.038** |
| | **95% CI** | 0.87–0.94 | 0.95–0.99 | 0.91–0.97 | 0.95–0.99 | 0.90–0.96 |
| RF | Fold-1 | 0.955 | 0.968 | 0.961 | 0.969 | 0.955 |
| | Fold-2 | 0.977 | 0.952 | 0.964 | 0.991 | 0.956 |
| | Fold-3 | 0.842 | 0.912 | 0.877 | 0.930 | 0.853 |
| | Fold-4 | 0.842 | 0.971 | 0.906 | 0.958 | 0.899 |
| | Fold-5 | 0.970 | 0.932 | 0.951 | 0.988 | 0.914 |
| | **Mean ± std** | **0.917 ± 0.069** | 0.947 ± 0.025 | 0.932 ± 0.039 | 0.967 ± 0.025 | 0.915 ± 0.043 |
| | **95% CI** | 0.86–0.98 | 0.92–0.97 | 0.90–0.97 | 0.95–0.99 | 0.88–0.95 |

**Note:** Input features included RNFL, GC-IPL and Macular thickness data in spatial and frequency domain. 95% confidence interval (CI) = Mean ± 1.96 × Standard Error (SE), SE= std / /$\sqrt{n}$, n=5, CI = Confidence Interval. The highlighted numbers represent highest performance among the folds. KNN: K-Nearest Neighbours, SVM: Support Vector Machines, RF: Random Forests.

**Supplementary Table 5:** Performance of the three different classification models for diagnosing glaucoma stages with 5-fold cross validation using artefact-free data without MICE-based imputation and SMOTE-based oversampling.

| | | One vs rest approach | | | One vs one approach | | |
|---|---|---|---|---|---|---|---|
| Classifier | Metric | Early vs rest | Moderate vs rest | Advanced vs rest | Early vs Normal | Moderate vs Normal | Advanced vs Normal |
| KNN | Sensitivity | **0.872 ± 0.083** | **0.896 ± 0.075** | 0.822 ± 0.151 | 0.762 ± 0.045 | 0.797 ± 0.066 | 0.886 ± 0.064 |
| | Specificity | 0.646 ± 0.031 | 0.727 ± 0.042 | 0.869 ± 0.022 | 0.954 ± 0.039 | 0.948 ± 0.035 | **1 ± 0** |
| | Accuracy | 0.759 ± 0.057 | 0.811 ± 0.059 | 0.845 ± 0.086 | 0.858 ± 0.030 | 0.872 ± 0.043 | 0.943 ± 0.032 |
| | AUC | 0.802 ± 0.018 | 0.839 ± 0.059 | 0.884 ± 0.071 | 0.877 ± 0.026 | 0.889 ± 0.036 | 0.943 ± 0.032 |
| | F1-Score | 0.790 ± 0.053 | 0.714 ± 0.068 | 0.813 ± 0.151 | 0.794 ± 0.056 | 0.816 ± 0.054 | 0.938 ± 0.034 |
| SVM | Sensitivity | 0.806 ± 0.171 | 0.717 ± 0.157 | 0.789 ± 0.194 | 0.866 ± 0.095 | 0.946 ± 0.087 | **1 ± 0** |
| | Specificity | 0.697 ± 0.059 | 0.788 ± 0.040 | 0.869 ± 0.028 | **0.970 ± 0.027** | **0.979 ± 0.017** | **1 ± 0** |
| | Accuracy | 0.752 ± 0.115 | 0.752 ± 0.099 | 0.829 ± 0.097 | **0.918 ± 0.049** | 0.962 ± 0.037 | **1 ± 0** |
| | AUC | 0.807 ± 0.069 | 0.788 ± 0.050 | 0.880 ± 0.054 | **0.954 ± 0.032** | 0.980 ± 0.033 | **1 ± 0** |
| | F1-Score | **0.798 ± 0.047** | 0.741 ± 0.068 | 0.806 ± 0.194 | **0.884 ± 0.066** | 0.927 ± 0.034 | **1 ± 0** |
| RF | Sensitivity | 0.843 ± 0.098 | 0.863 ± 0.132 | **0.889 ± 0.141** | **0.921 ± 0.072** | **0.973 ± 0.060** | 1 ± 0 |
| | Specificity | **0.727 ± 0.051** | **0.788 ± 0.029** | **0.909 ± 0.022** | 0.899 ± 0.050 | 0.973 ± 0.022 | 0.997 ± 0.007 |
| | Accuracy | **0.785 ± 0.049** | **0.826 ± 0.066** | **0.899 ± 0.070** | 0.910 ± 0.045 | **0.973 ± 0.029** | 0.998 ± 0.003 |
| | AUC | **0.849 ± 0.024** | **0.871 ± 0.046** | **0.951 ± 0.022** | 0.947 ± 0.043 | **0.987 ± 0.021** | **1 ± 0** |
| | F1-Score | 0.728 ± 0.040 | **0.834 ± 0.052** | **0.902 ± 0.109** | 0.843 ± 0.050 | **0.937 ± 0.044** | 0.987 ± 0.030 |
| AVERAGE | Sensitivity | 0.840 ± 0.098 | 0.825 ± 0.132 | 0.833 ± 0.141 | 0.850 ± 0.071 | 0.905 ± 0.071 | 0.962 ± 0.021 |
| | Specificity | 0.690 ± 0.051 | 0.768 ± 0.029 | 0.882 ± 0.022 | 0.941 ± 0.039 | 0.967 ± 0.025 | 0.999 ± 0.002 |
| | Accuracy | 0.765 ± 0.049 | 0.796 ± 0.066 | 0.858 ± 0.070 | 0.896 ± 0.041 | 0.936 ± 0.036 | 0.980 ± 0.012 |
| | AUC | 0.819 ± 0.024 | 0.833 ± 0.046 | 0.905 ± 0.022 | 0.926 ± 0.034 | 0.952 ± 0.030 | 0.981 ± 0.011 |
| | F1-Score | 0.705 ± 0.040 | 0.896 ± 0.052 | 0.954 ± 0.109 | 0.840 ± 0.057 | 0.893 ± 0.044 | 0.975 ± 0.021 |

**Note:** The reported numbers are mean ± standard deviation over the 5 folds. Input features included RNFL, GC-IPL and Macular thickness features. Advanced glaucoma cases included are only based on mean deviation of visual fields. The highlighted numbers represent highest performance (for each metric) among the classifiers. KNN: K-Nearest Neighbours, SVM: Support Vector Machines, RF: Random Forests

**Supplementary Table 6:** Performance of the different classifiers in distinguishing overall glaucoma patients versus normal in the different folds of the five-fold cross-validation experiment (using artefact-free data without imputation and oversampling).

| Approach | Fold | Sensitivity | Specificity | Accuracy | AUC | F1-Score |
|---|---|---|---|---|---|---|
| KNN | Fold-1 | 0.897 | 0.932 | 0.914 | 0.929 | 0.868 |
| | Fold-2 | 0.778 | 0.948 | 0.863 | 0.906 | 0.843 |
| | Fold-3 | 0.850 | 0.871 | 0.860 | 0.884 | 0.845 |
| | Fold-4 | 0.892 | 0.892 | 0.892 | 0.941 | 0.879 |
| | Fold-5 | 0.839 | 0.986 | 0.912 | 0.942 | 0.897 |
| | **Mean ± std** | 0.851 ± 0.048 | 0.926 ± 0.046 | 0.888 ± 0.026 | 0.921 ± 0.025 | 0.866 ± 0.023 |
| | **95% CI** | 0.81–0.89 | 0.89–0.97 | 0.87–0.91 | 0.9–0.94 | 0.85–0.89 |
| SVM | Fold-1 | 0.966 | 0.932 | 0.949 | 0.992 | 0.929 |
| | Fold-2 | 0.844 | 0.966 | 0.905 | 0.93 | 0.894 |
| | Fold-3 | 0.950 | 0.968 | 0.959 | 0.983 | 0.950 |
| | Fold-4 | 0.892 | 1 | 0.946 | 0.977 | 0.943 |
| | Fold-5 | 0.903 | 0.972 | 0.938 | 0.964 | 0.918 |
| | **Mean ± std** | 0.911 ± 0.048 | **0.968 ± 0.024** | **0.939 ± 0.021** | 0.969 ± 0.024 | **0.927 ± 0.022** |
| | **95% CI** | 0.87–0.95 | 0.95–0.99 | 0.92–0.96 | 0.95–0.99 | 0.91–0.95 |
| RF | Fold-1 | 1 | 0.932 | 0.966 | 0.990 | 0.921 |
| | Fold-2 | 0.844 | 0.897 | 0.870 | 0.933 | 0.861 |
| | Fold-3 | 0.950 | 0.919 | 0.935 | 0.973 | 0.916 |
| | Fold-4 | 0.919 | 0.969 | 0.944 | 0.983 | 0.932 |
| | Fold-5 | 0.903 | 0.972 | 0.938 | 0.967 | 0.918 |
| | **Mean ± std** | **0.923 ± 0.058** | 0.938 ± 0.032 | 0.931 ± 0.036 | **0.969 ± 0.022** | 0.909 ± 0.028 |
| | **95% CI** | 0.87–0.97 | 0.91–0.97 | 0.9–0.96 | 0.95–0.99 | 0.88–0.93 |

**Note:** Input features included RNFL, GC-IPL and Macular thickness data in spatial and frequency domain. 95% confidence interval (CI) = Mean ± 1.96 × Standard Error (SE), SE= std/$\sqrt{n}$, n=5, CI = Confidence Interval. The highlighted numbers represent highest performance among the folds. KNN: K-Nearest Neighbours, SVM: Support Vector Machines, RF: Random Forests.

**Supplementary Table 7:** Comparative performance for human (clinicians) vs machine (AI-based models) for diagnosing glaucoma; the bolded numbers were used to compare the overall performance in terms of accuracy

| Decision maker | Stages | Sensitivity | Specificity | Accuracy | F1-Score |
|---|---|---|---|---|---|
| Optometrist-1 (Unmasked-sequential) | Overall Glaucoma | 0.871 | 0.855 | 0.863 | 0.865 |
| | Early | 0.650 | 0.878 | 0.825 | 0.634 |
| | Moderate | 0.833 | 0.966 | 0.944 | 0.833 |
| | Advanced | 1 | 0.983 | 0.985 | 0.952 |
| Clinician-2 (Unmasked-sequential) | Overall Glaucoma | 0.900 | 0.800 | 0.853 | 0.867 |
| | Early | 0.714 | 0.835 | 0.806 | 0.638 |
| | Moderate | 0.818 | 0.949 | 0.928 | 0.782 |
| | Advanced | 1 | 1 | 1 | 1 |
| Clinician-3 (Unmasked-sequential) | Overall Glaucoma | 0.851 | 0.791 | 0.822 | 0.834 |
| | Early | 0.590 | 0.857 | 0.788 | 0.590 |
| | Moderate | 0.800 | 0.931 | 0.911 | 0.727 |
| | Advanced | 1 | 1 | 1 | 1 |
| Mean Clinician Performance (unmasked-sequential) | Overall Glaucoma | 0.874 | 0.815 | **0.846** | 0.855 |
| | Early | 0.651 | 0.857 | **0.806** | 0.621 |
| | Moderate | 0.817 | 0.949 | **0.928** | 0.781 |
| | Advanced | 1 | 0.994 | **0.995** | 0.984 |
| Clinician-1 (Unmasked-random) | Overall Glaucoma | 0.900 | 0.661 | 0.782 | 0.807 |
| | Early | 0.625 | 0.681 | 0.670 | 0.425 |
| | Moderate | 0.916 | 0.957 | 0.949 | 0.88 |
| | Advanced | 1 | 1 | 1 | 1 |

**Supplementary Table 8**: Comparative performance with other studies for human (clinicians) vs machine (AI-based models using RNFL, GC-IPL and Macular thickness data) for diagnosing glaucoma.

| Decision maker | Stages | Sensitivity | Specificity | Accuracy | AUC |
|---|---|---|---|---|---|
| Human | Early | 61.9%[4] (MD), 62.9% [4] (HAP) | 95.1%[4] (MD), 87.4%[4] (HAP) | - | - |
| | Moderate | 66.2%[4] (MD), 85.9% (HAP) | 95.1%[4] (MD), 87.4%[4] (HAP) | - | - |
| | Advanced | 96.8%[4] (MD), 96.8% (HAP) | 95.1%[4], 87.4%[4] (HAP) | - | - |
| | Overall Glaucoma | 74.7%[4] , 75.6%[5], 92%[6] | 87.4%[4], 77.8%[5], 74%[6] | 80.5%[4], 80%[6] | |
| Machine | Early (RF) | 91.3% | 88.7% | 89.9% | 0.93 |
| | Moderate (RF) | 95.7% | 92.4% | 94% | 0.97 |
| | Advanced (RF) | 100% | 97.8% | 98.9% | 0.99 |
| | Overall Glaucoma (RF) | 90.5% | 91.4% | 90.9% | 0.95 |
| | Overall Glaucoma (SVM) | 90.3% | 93.8% | 92.2% | 0.96 |

**Note:** Hodapp-Anderson-Parrish scale (HAP), mean deviation (MD) as reference while considering glaucoma stages, RF: Random Forests, SVM: Support Vector Machine

# Supplementary Figures

(a)

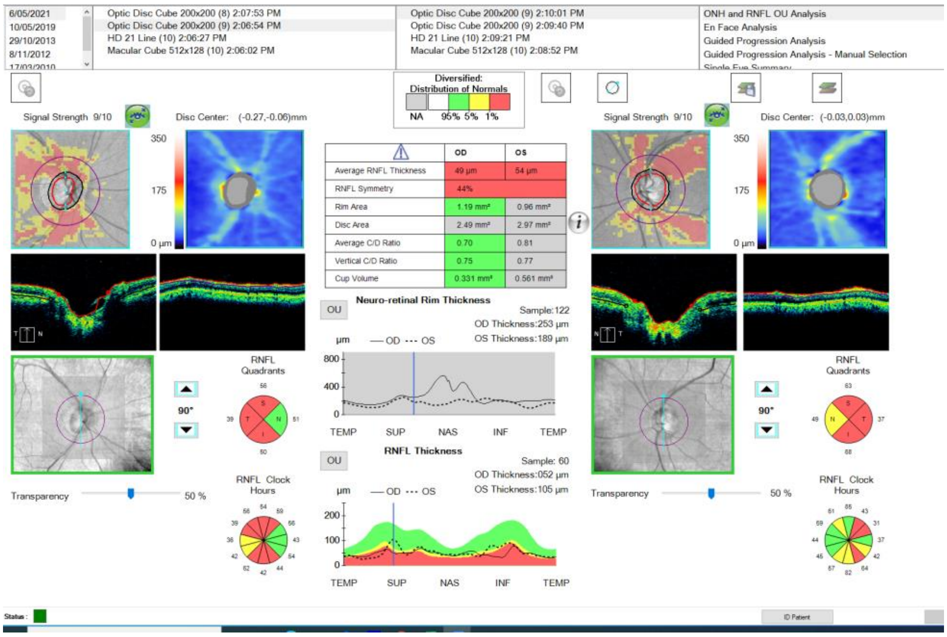


(b)

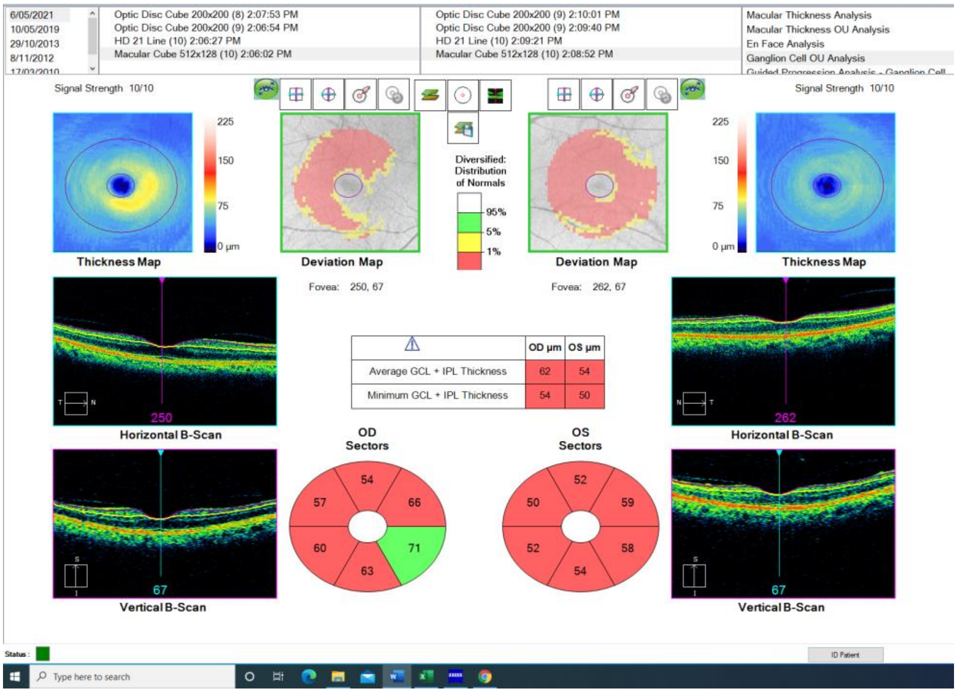

(c)

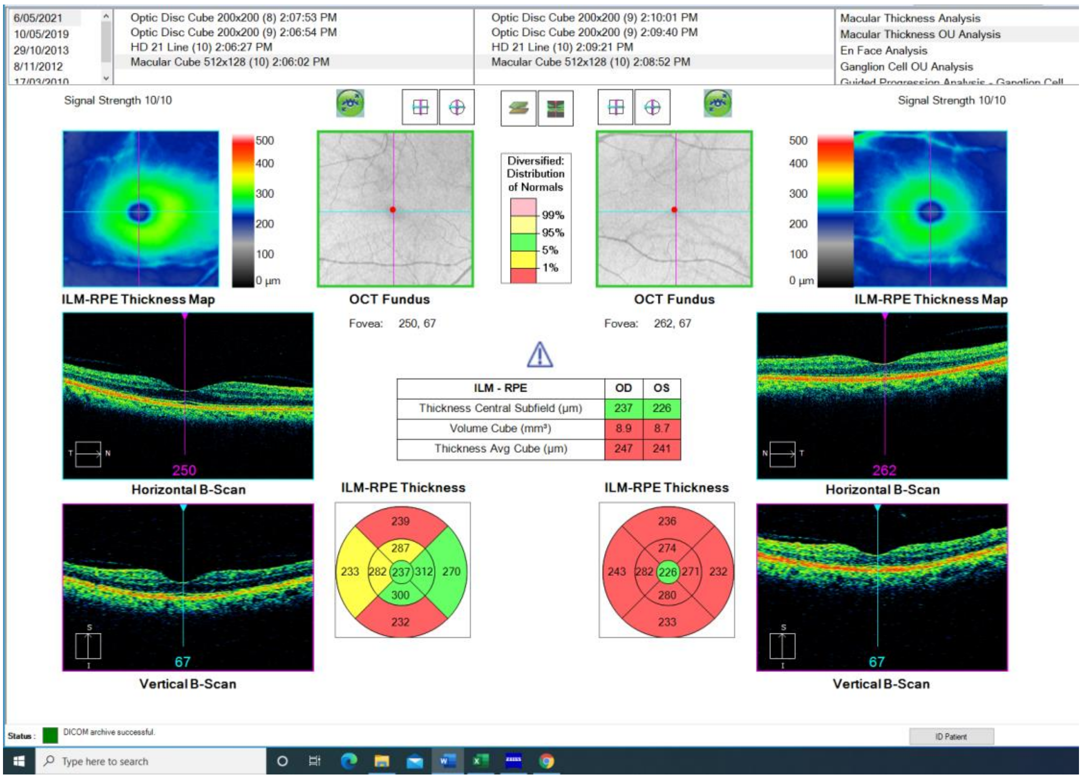


**Supplementary Figure 1:** Example of CIRRUS OCT based analysis (a) RNFL (b) GC-IPL (c) MC thickness analysis.

(a)

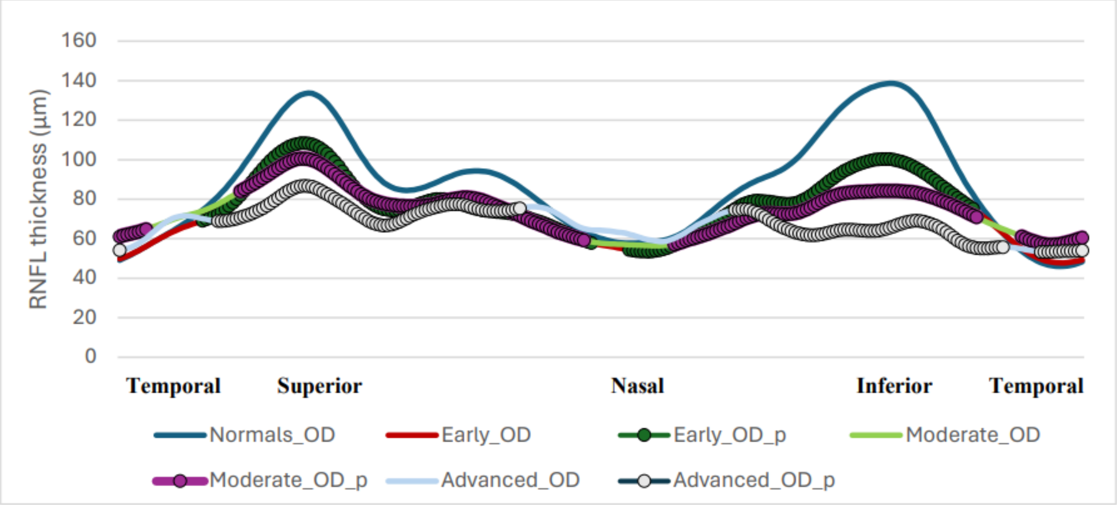


(b)

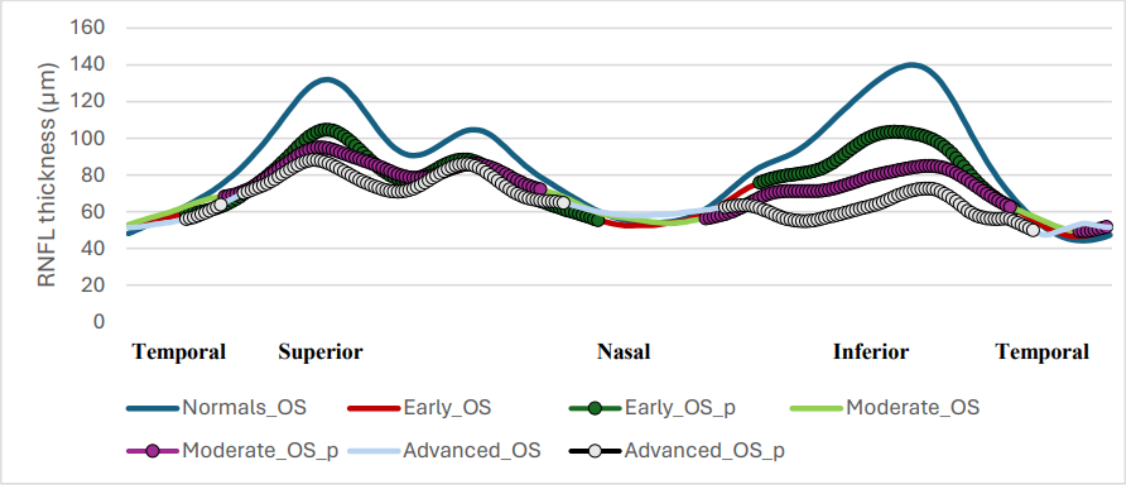


**Supplementary Figure 2:** TSNIT plots for glaucoma patients with different severity levels. (a) Right eye (*oculus dexter*, *OD*). (b) Left eye (*oculus sinister*, *OS*). The disc-like dots area (labelled as 'p') represents the points having significant difference ($p<0.05$) from the normal. Advanced glaucoma cases included are only based on mean deviation of visual fields.

(a) RNFL thickness (average)

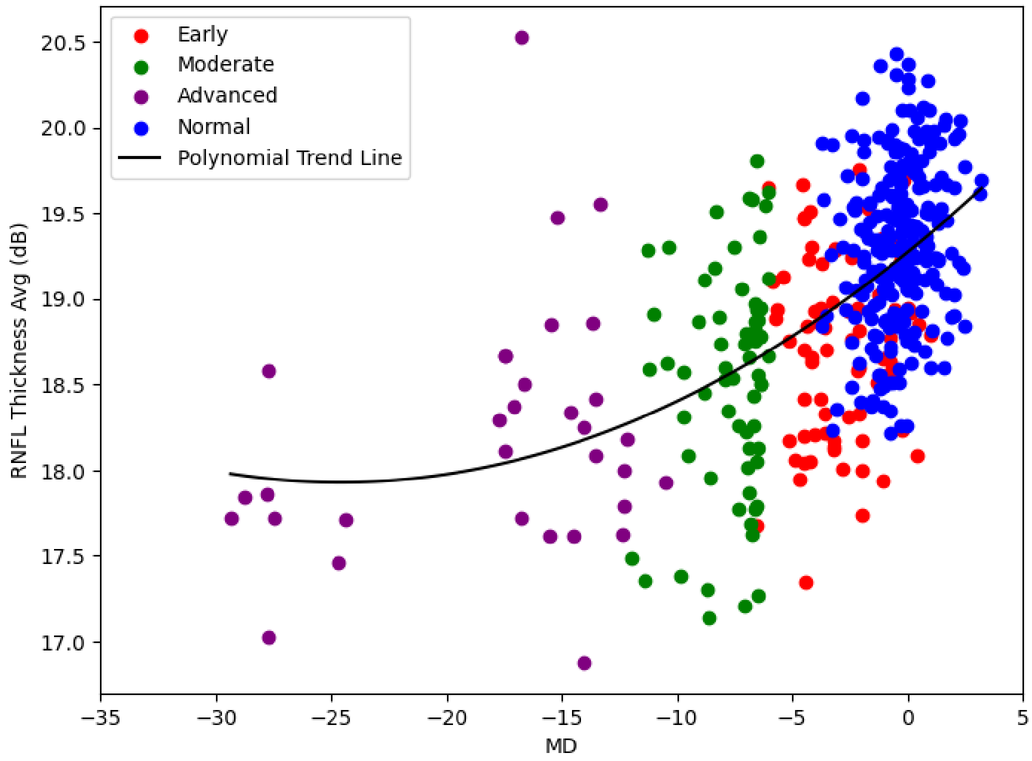


**(b)** RNFL inferior (mean)

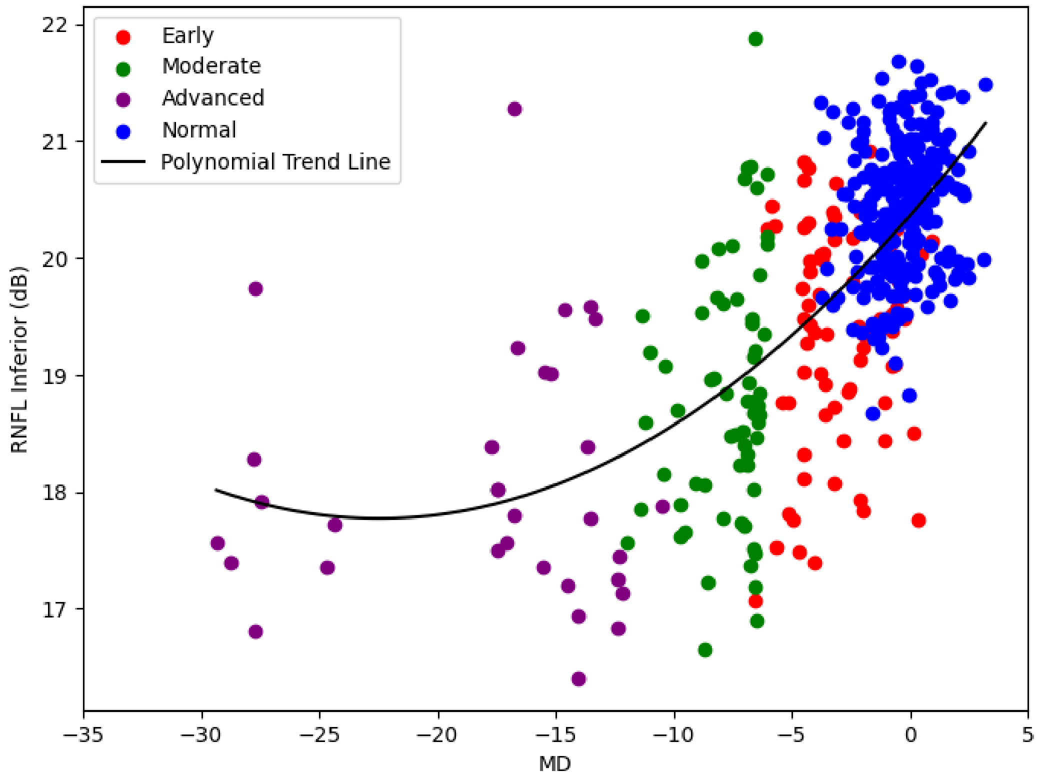

(c) RNFL superior (mean)

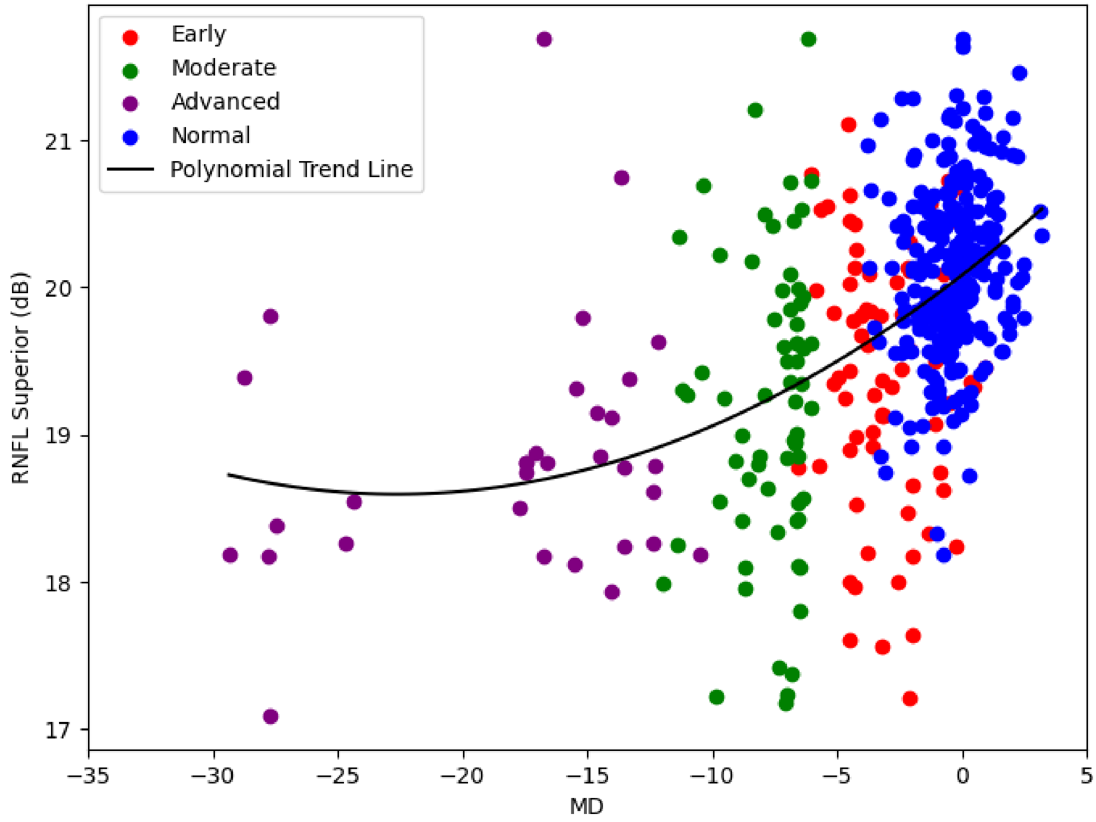


**(d)** CG-IPL thickness (average)

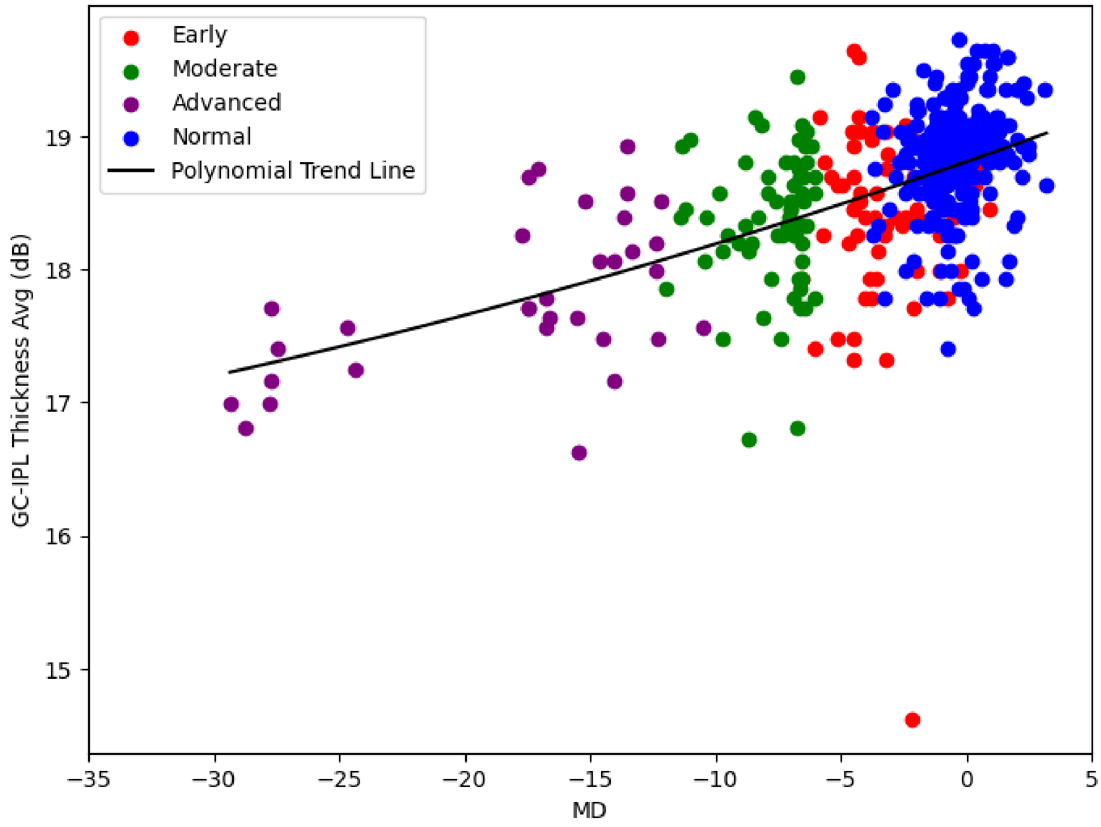

**(e)** CG-IPL inferior thickness

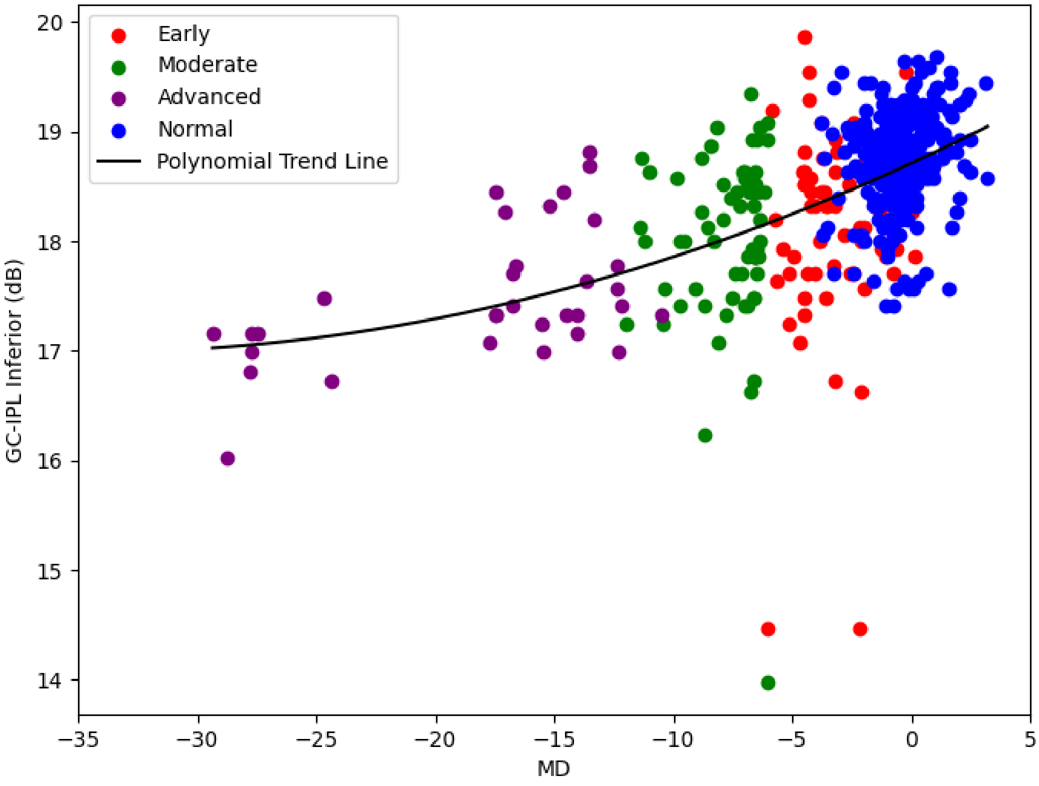


**(f)** GC-IPL inferotemporal

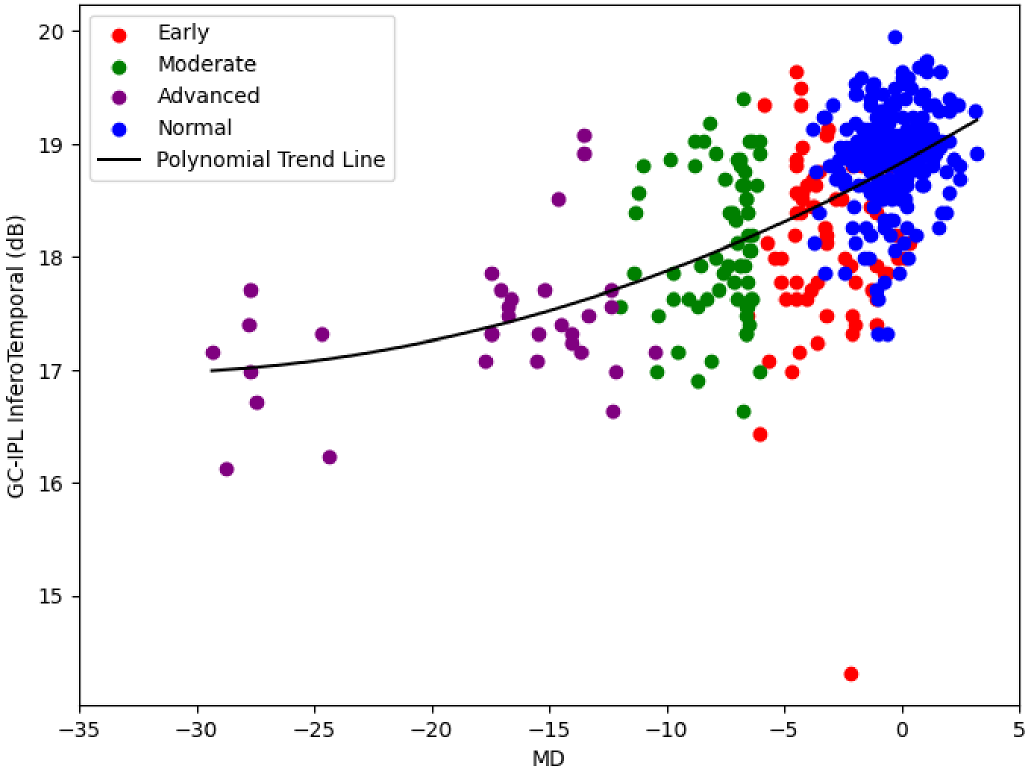

(g) GC-IPL superotemporal

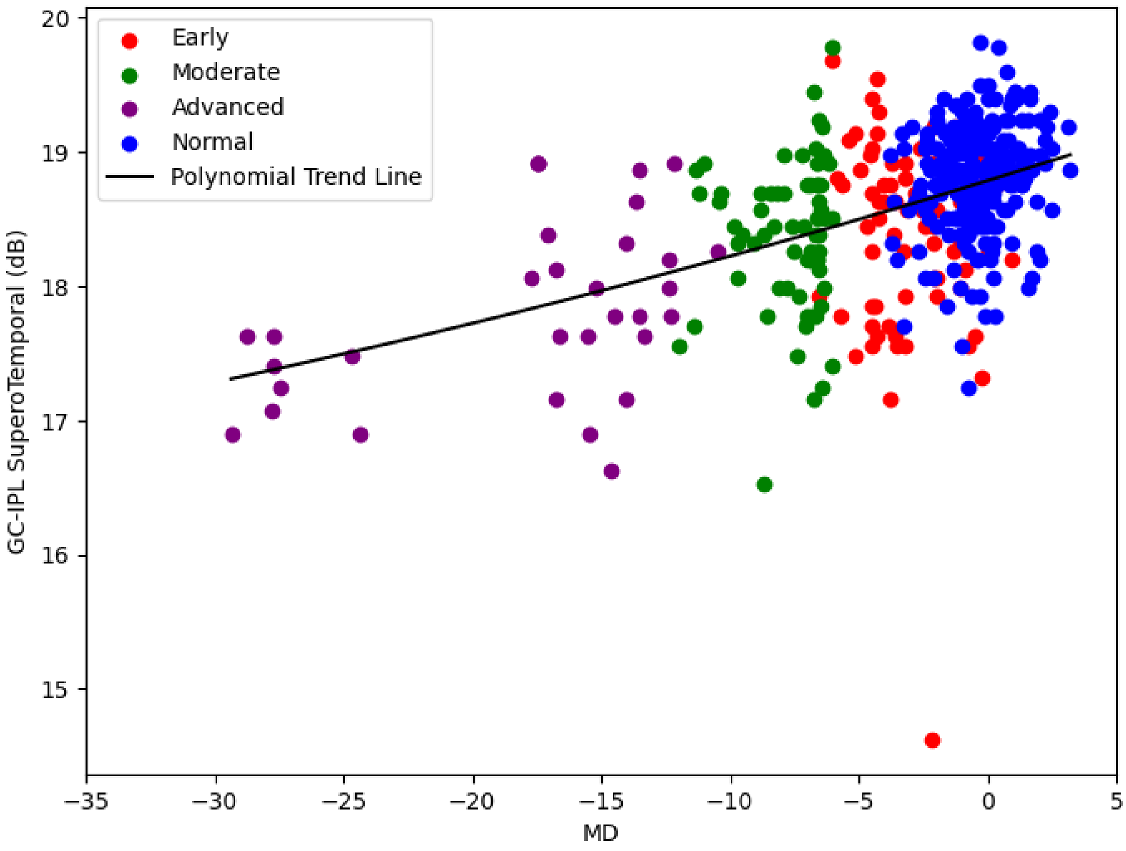


**Supplementary Figure 3:** Scatter plots of the RNFL/GC-IPL thickness versus MD of visual fields to visualise the structural-functional relationships.

(a) One vs Rest (OvR)

### K nearest neighbours (KNN)

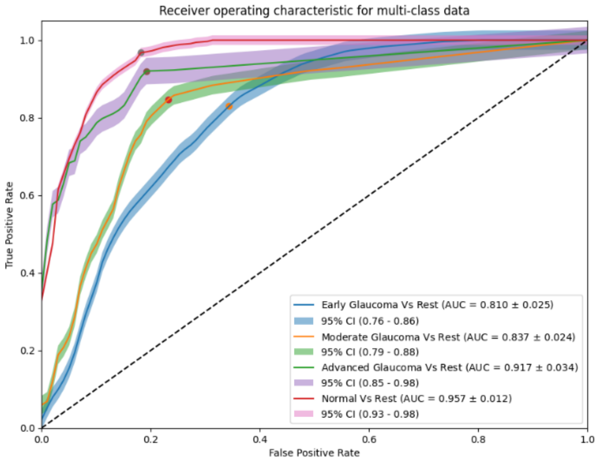


### Support vector machines (SVM)

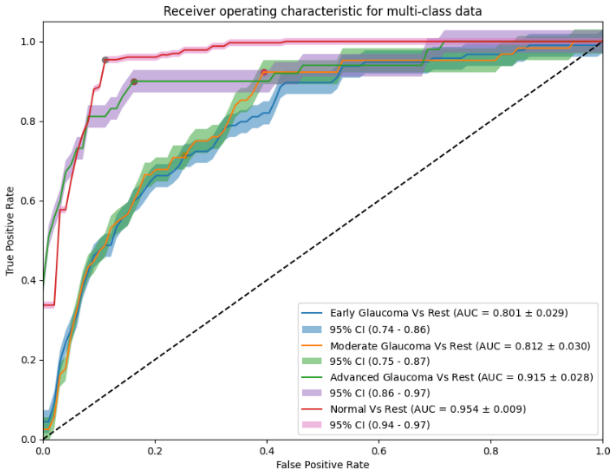


### Random forest (RF)

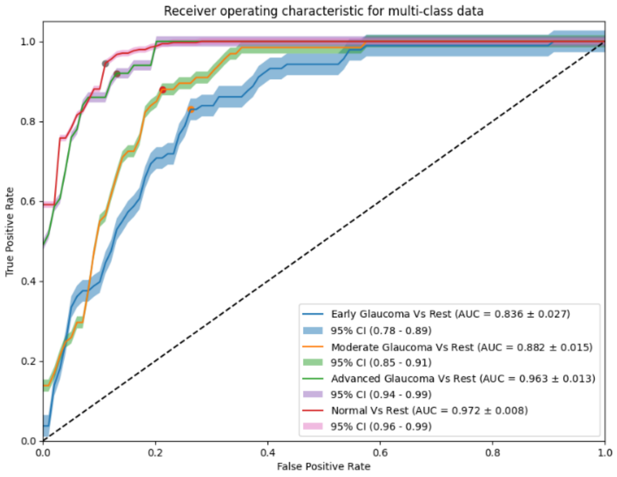

(b) One vs One (OvO)

**K nearest neighbours (KNN)** **Support vector machines (SVM)** **Random forest (RF)**

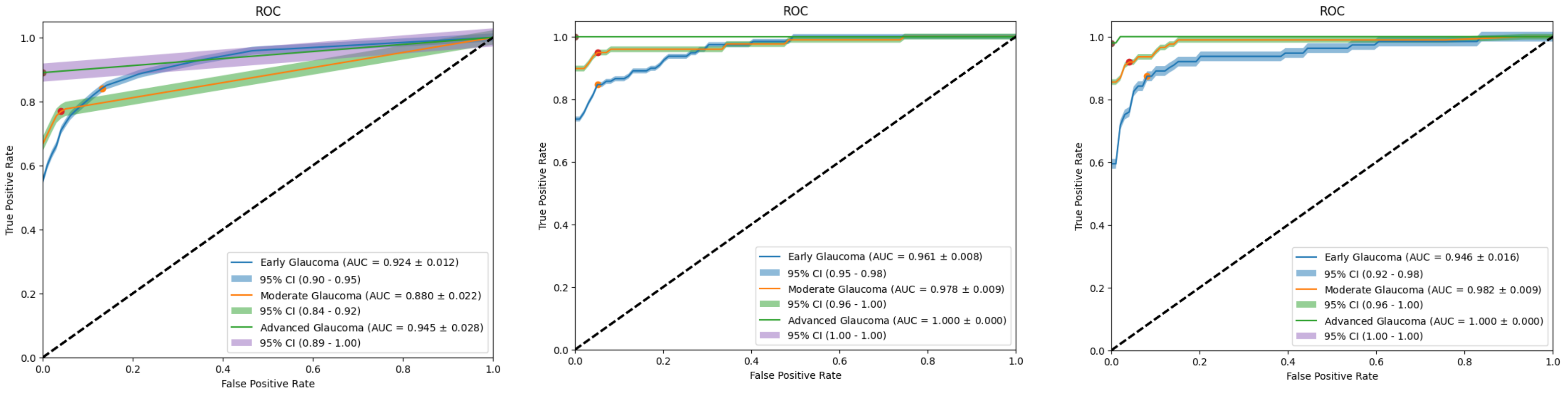


**Supplementary Figure 4:** Performance of glaucoma stages diagnosis (using RNFL, GC-IPL and MC data) (a) One vs Rest and (b) One vs One approach. Reported AUC: Mean ± standard error. standard error = standard deviation/ $\sqrt{n}$ , n=5 (for five-fold cross validation).

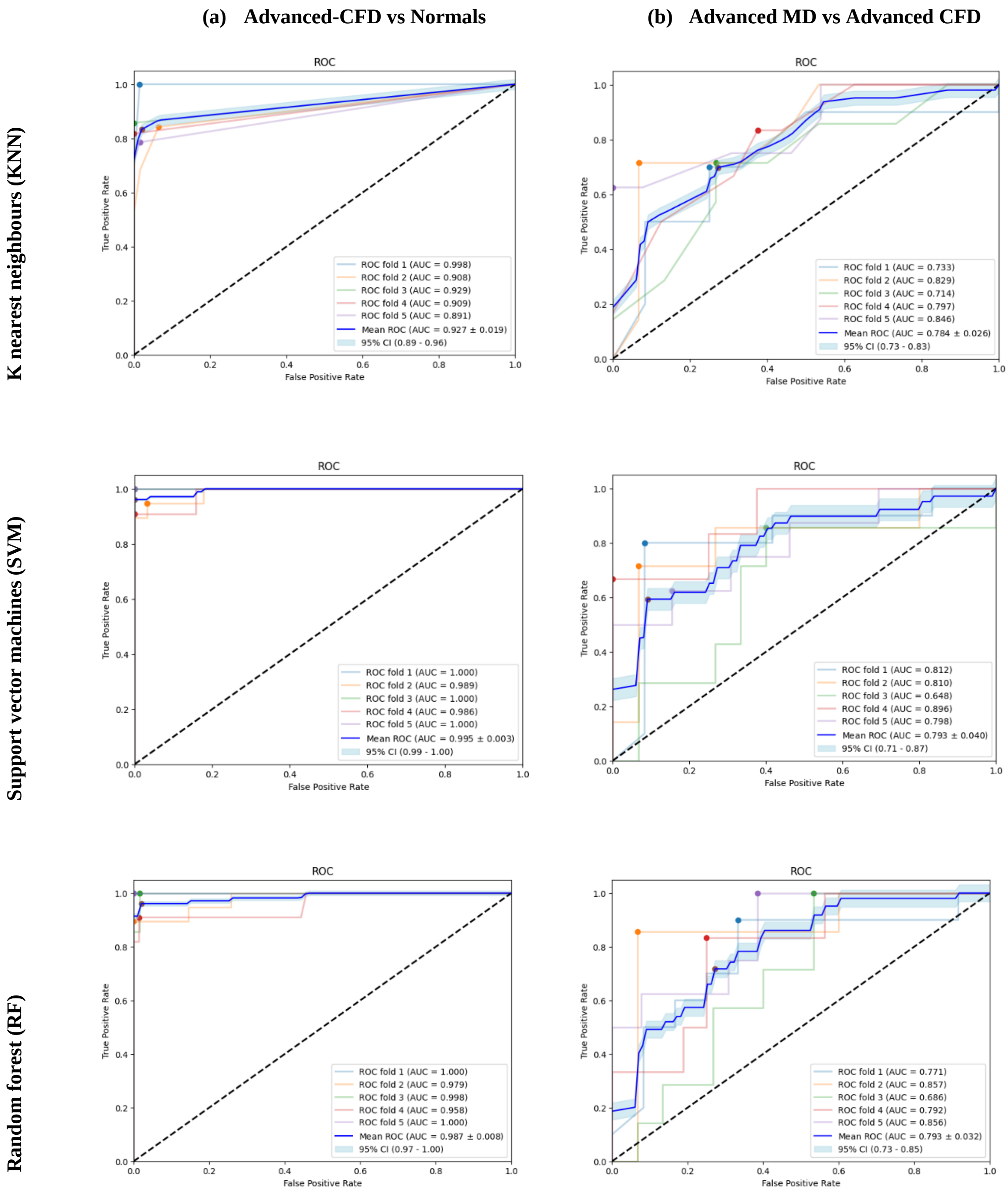


**Supplementary Figure 5:** Performance of differential diagnosis of advanced glaucoma based on CFD (using RNFL, GC-IPL and MC data). Reported AUC: Mean ± standard error. standard error = standard deviation/ $\sqrt{n}$ , n=5 (for five-fold cross validation)

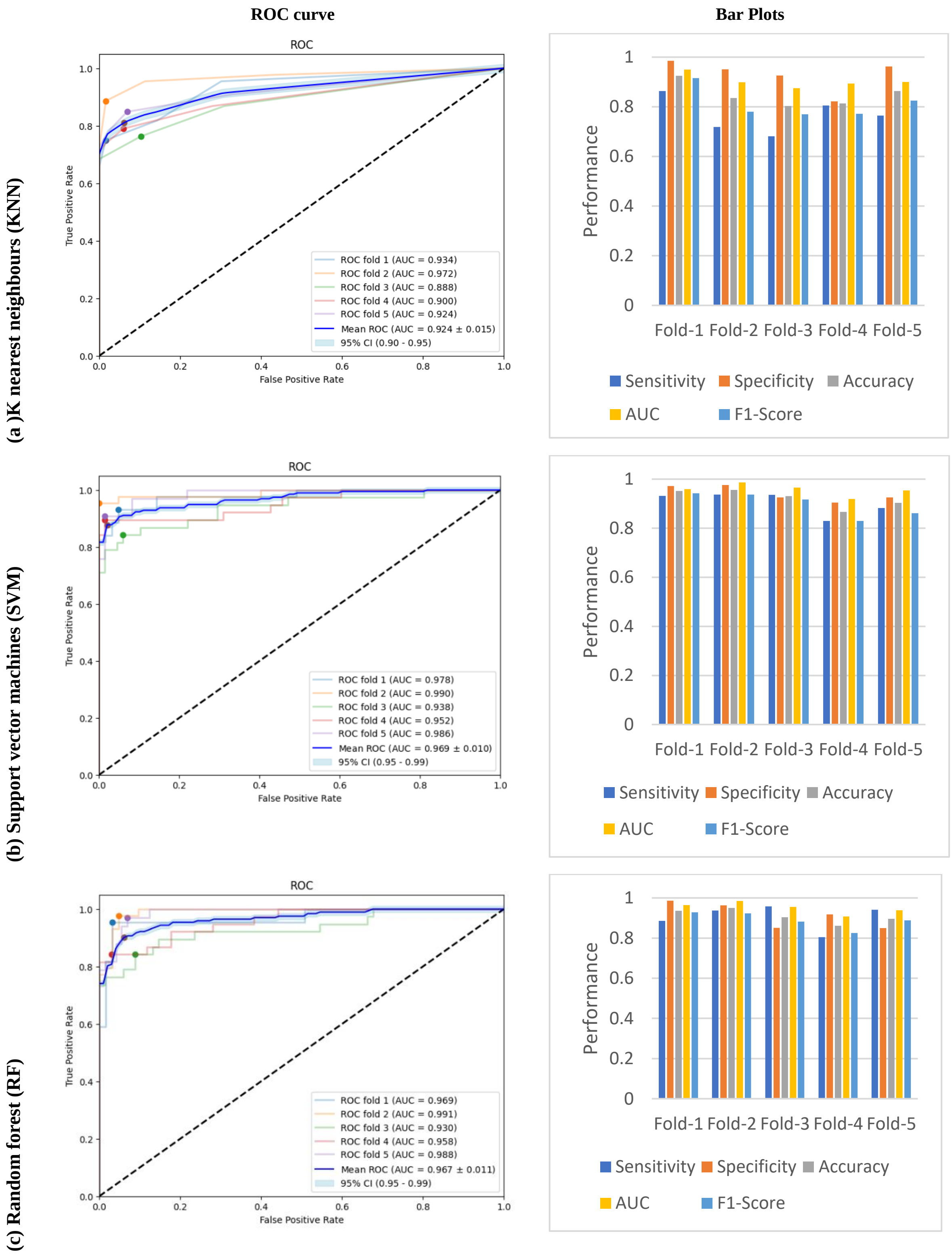


**Supplementary Figure 6:** Performance of differential diagnosis of overall glaucoma and normal (using RNFL, GC-IPL and MC data). Reported AUC: Mean ± standard error. standard error = standard deviation/ $\sqrt{n}$ , n=5 (for 5-fold cross validation).

***(a)** A sample of a coded patient with masked RNFL provided to the optometrists for grading.*

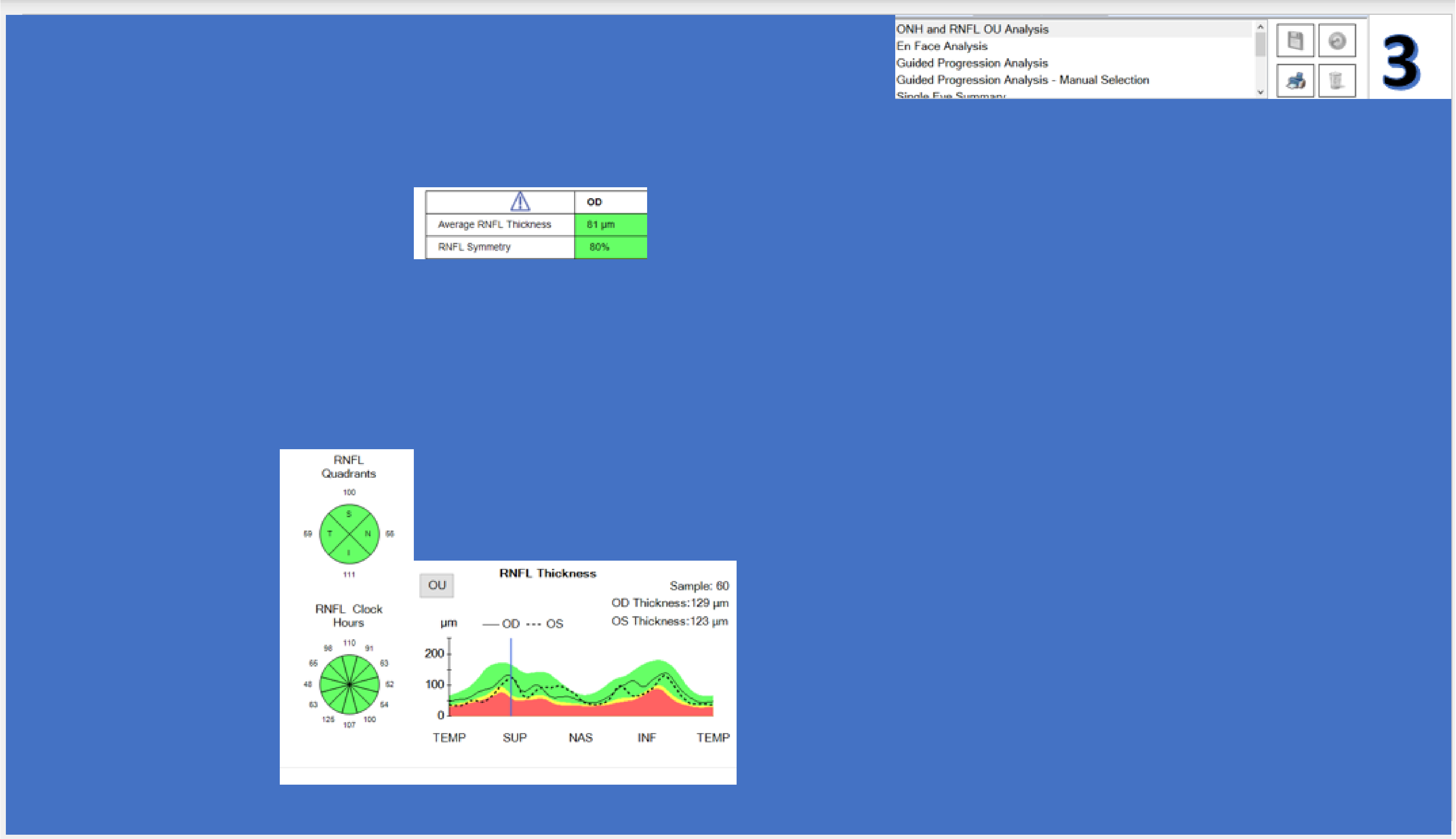

***(b)*** *A sample of unmasked RNFL provided to the optometrists for grading sequentially (unmasked- sequential).*

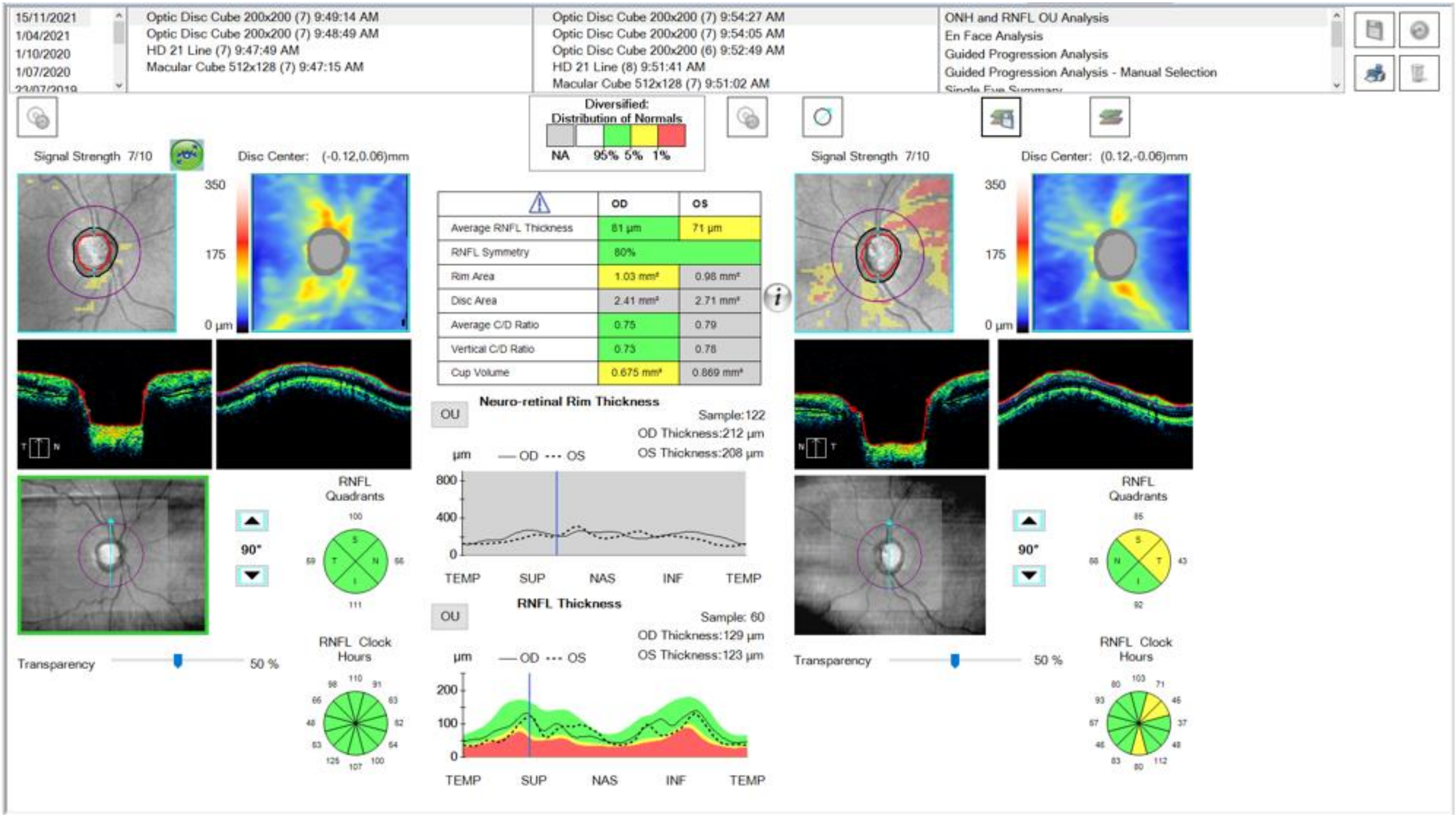

*(c) A sample of unmasked RNFL provided to the optometrists for grading randomly (unmasked-random)*

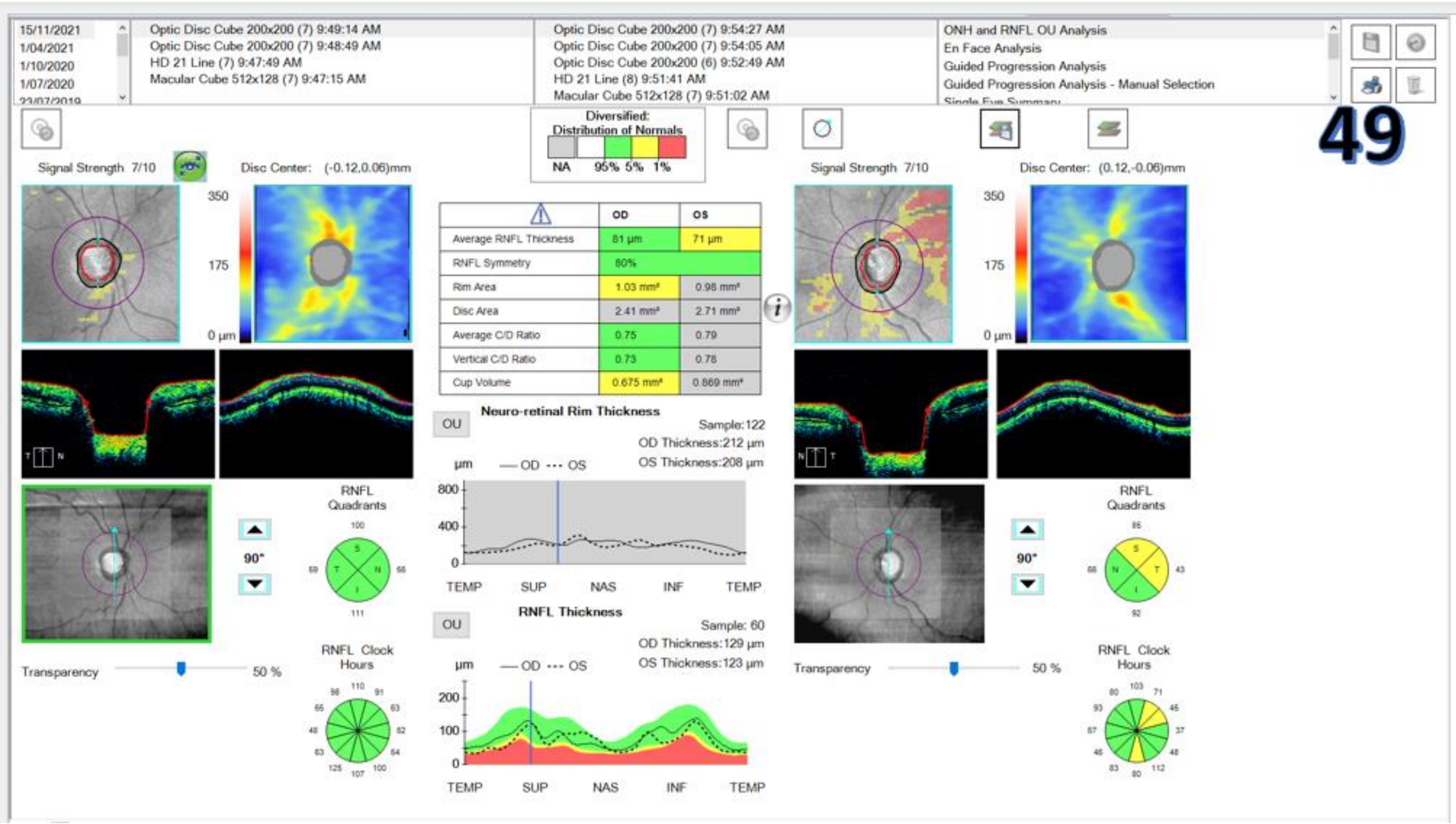


**Supplementary Figure 7:** A sample of (a) masked (b)unmasked sequential and (c) unmasked random RNFL data provided to the optometrists for grading randomly (unmasked-random)

*(a)*

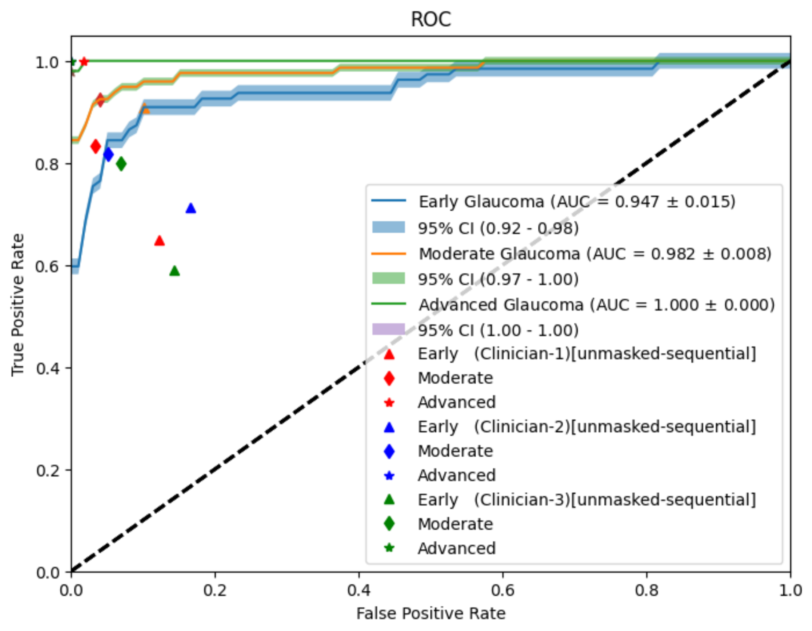


*(b)*

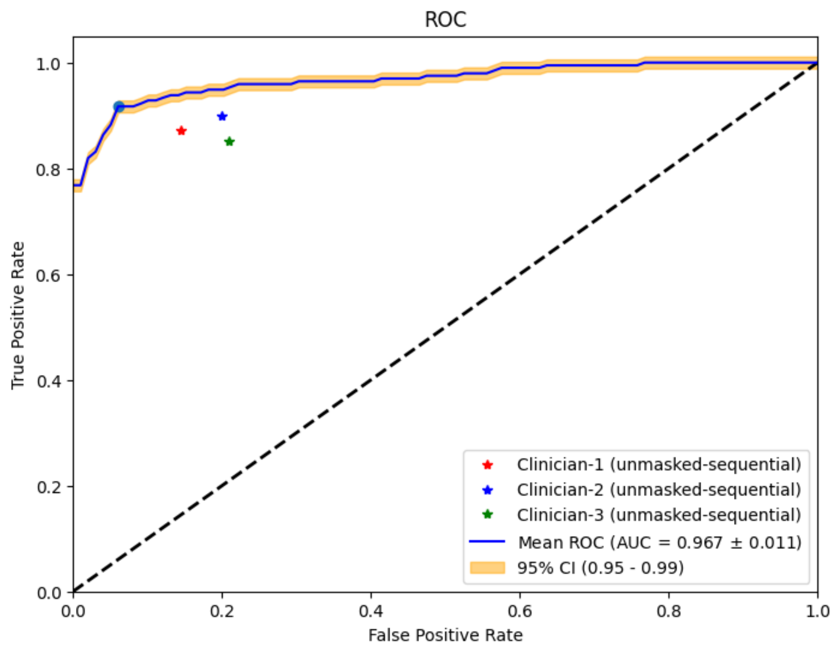


**Supplementary Figure 8:** Clinician's performance for sequentially unmasked data: (a) glaucoma staging (b) overall glaucoma diagnosis. Reported AUC: Mean ± standard error. standard error = standard deviation/ $\sqrt{n}$ , n=5 (for 5-fold cross validation).

*(a)*

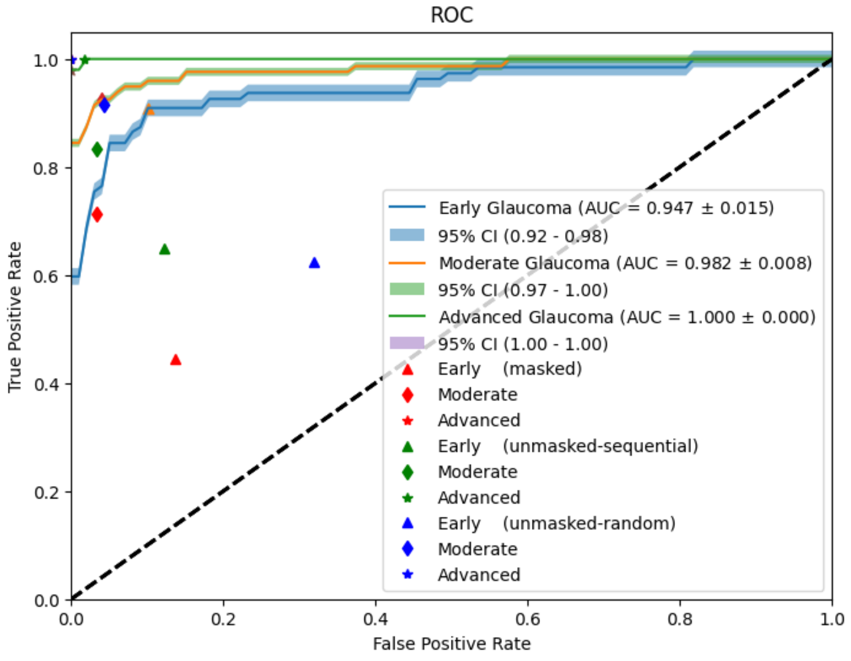


*(b)*

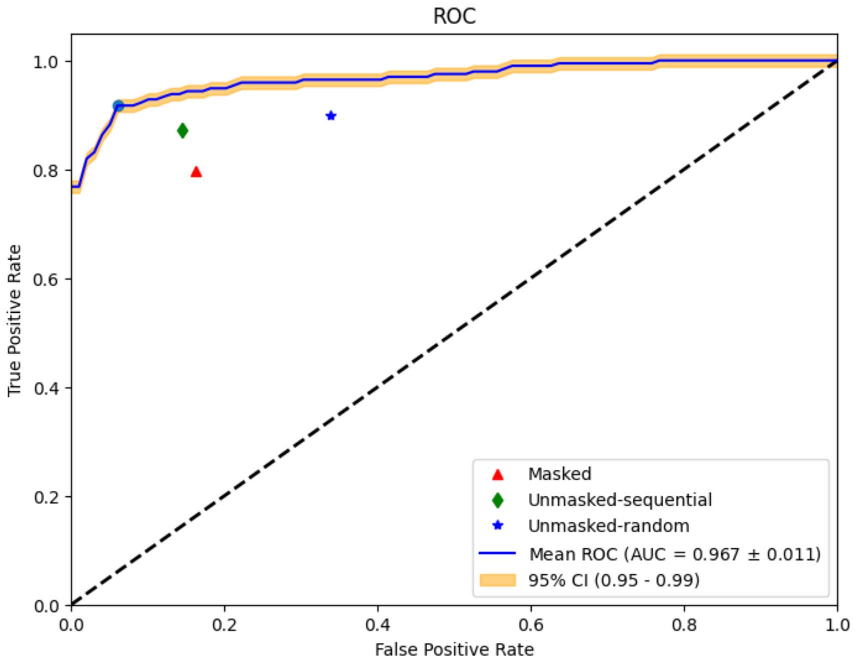


**Supplementary Figure 9**: Clinicians' performance with masked, unmasked sequential and unmasked random dataset vs machine performance (RF): (a) glaucoma staging (b) overall glaucoma diagnosis. Reported AUC: Mean ± standard error. standard error = standard deviation/ $\sqrt{n}$ , n=5 (for 5-fold cross validation).

**PDP & ICE**

**Estimating decision boundary**

(a) RNFL Symmetry

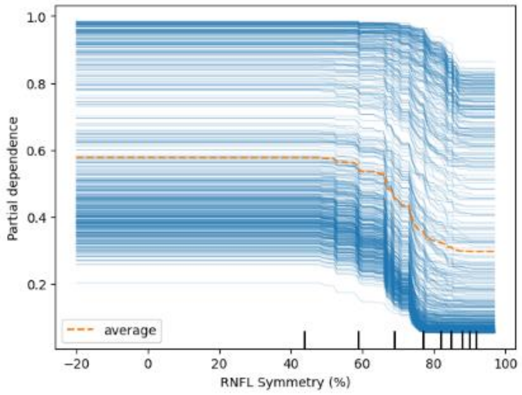


(b) RNFL Symmetry, cut-off=71%

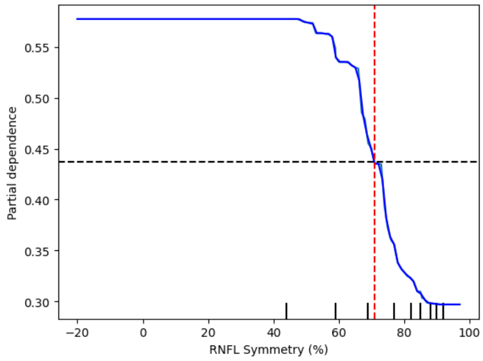


(c) RNFL Inferior

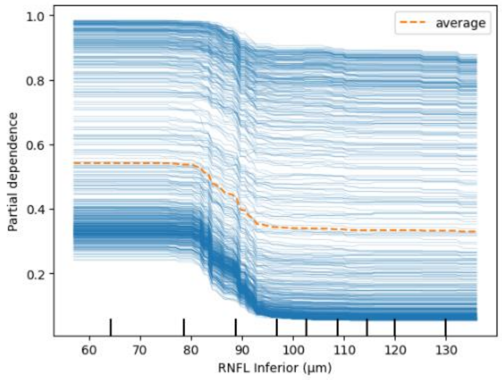


(d) RNFL Inferior, cut-off= 88.9 μm

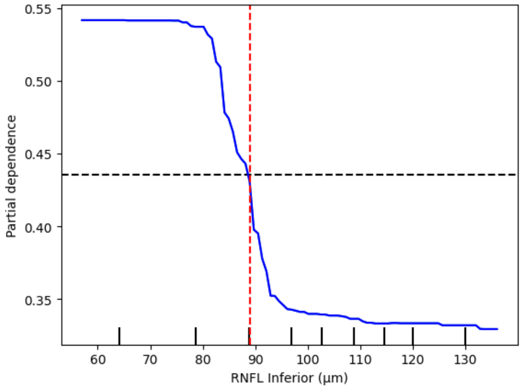


(e) RNFL Superior

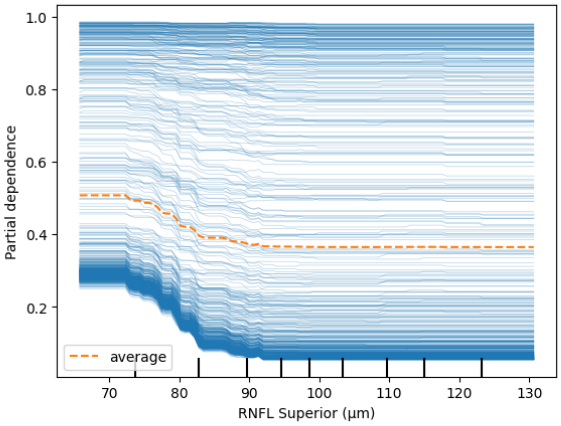


(f) RNFL Superior, cut-off=79.54 μm

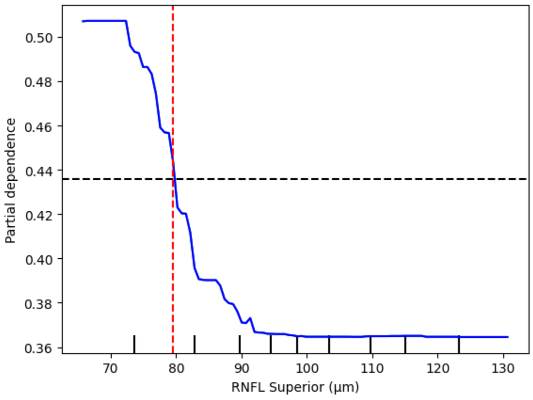

**PDP & ICE** | **Estimating decision boundary**

(g) Inferotemporal (IT) GC-IPL

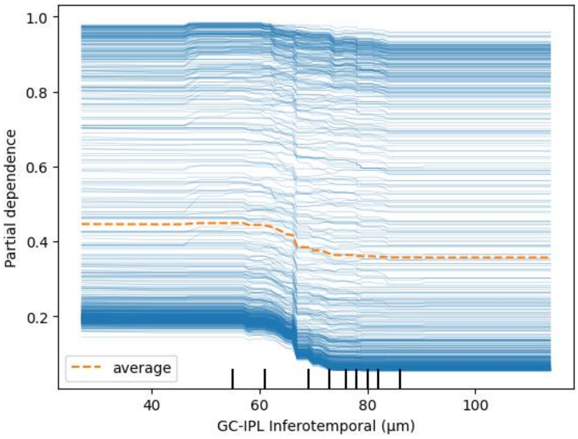


(h) GC-IPL Inferotemporal, cut-off=66.54 μm

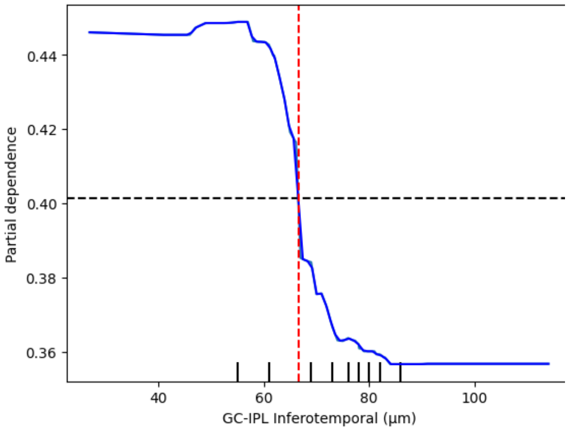


(i) Superotemporal (ST) GC-IPL

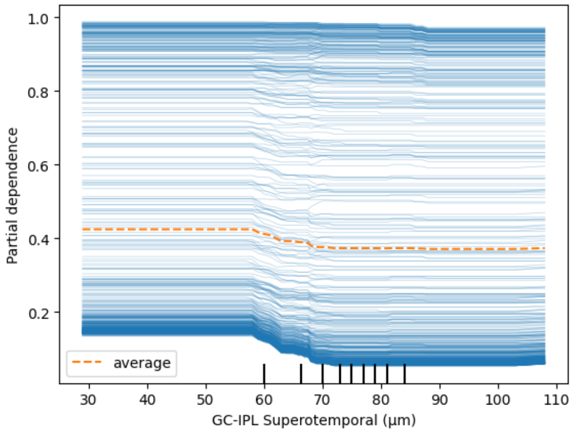


(j) GC-IPL Superotemporal, cut-off=62.52 μm

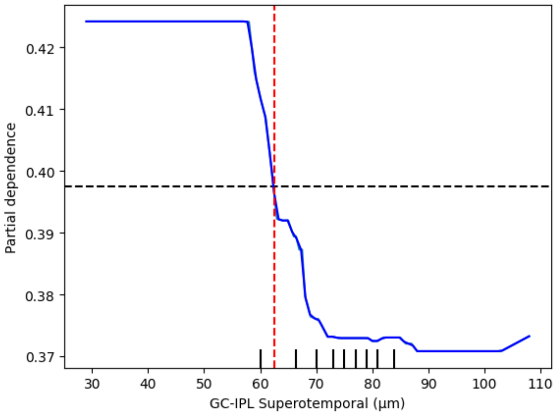


(k) Inferior GC-IPL

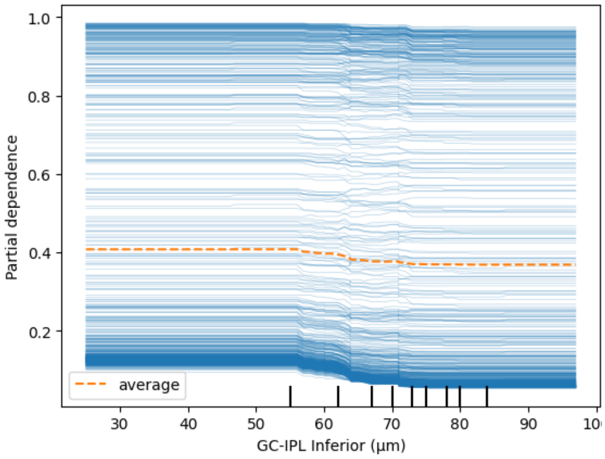


(l) GC-IPL Inferior, cut-off=63.54 μm

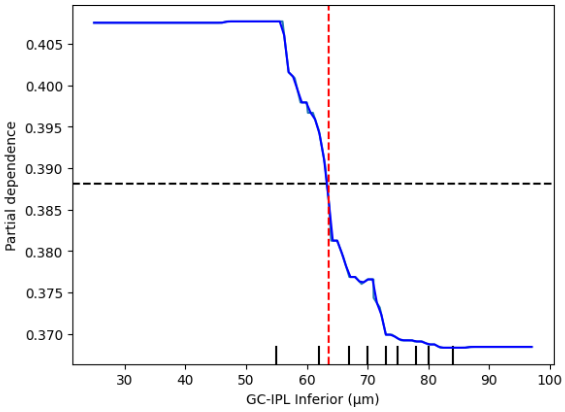

**PDP & ICE** | **Estimating decision boundary**

(m)Superior GC-IPL

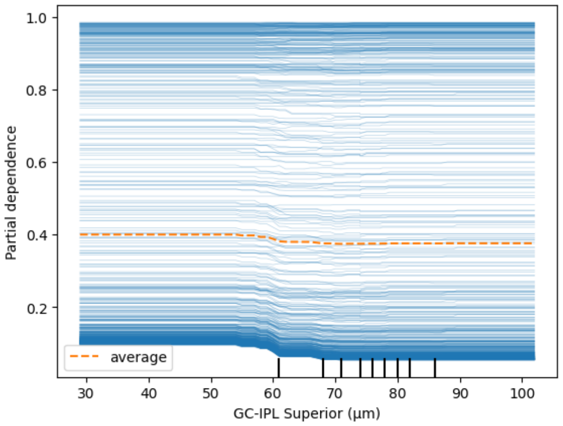


(n) Superior GC-IPL, cut-off=59.99 µm

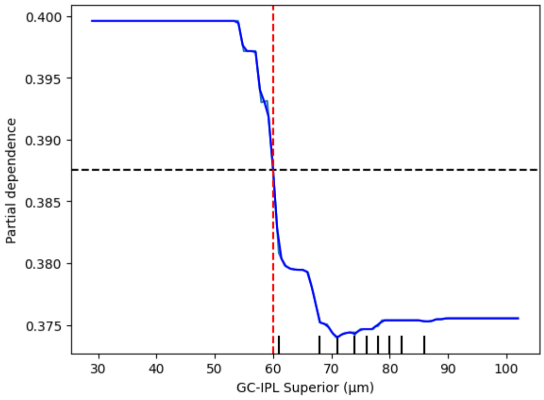


(o) Average RNFL

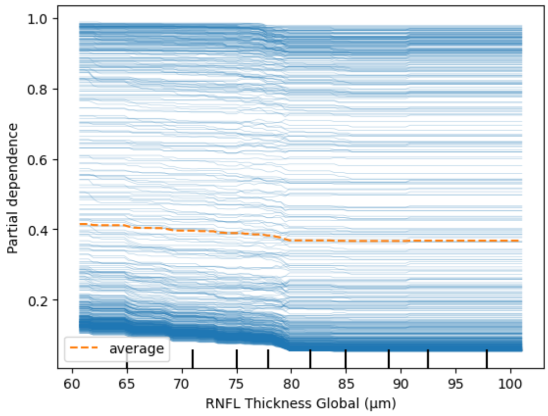


(p) RNFL thickness avg, cut-off= 71.0 µm

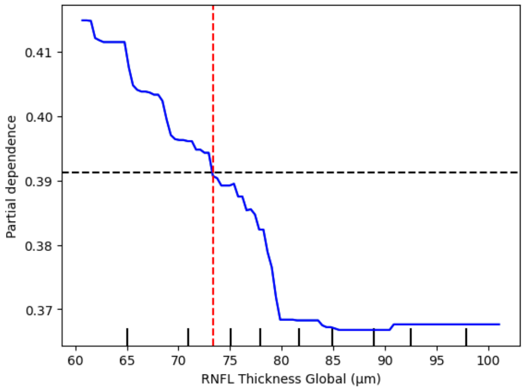


**Supplementary Figure 10:** and Individual Conditional Expectation (ICE) Plots (left column) and Partial dependency plot (PDP) (right columns) for major and minor features identified by SHAP. Based on the predicted probability of each feature by PDPs, a horizontal dashed line was drawn at the decision boundary and thus cut-off values were estimated at the intersection point of the decision boundary and the PDPs (vertical dashed line).

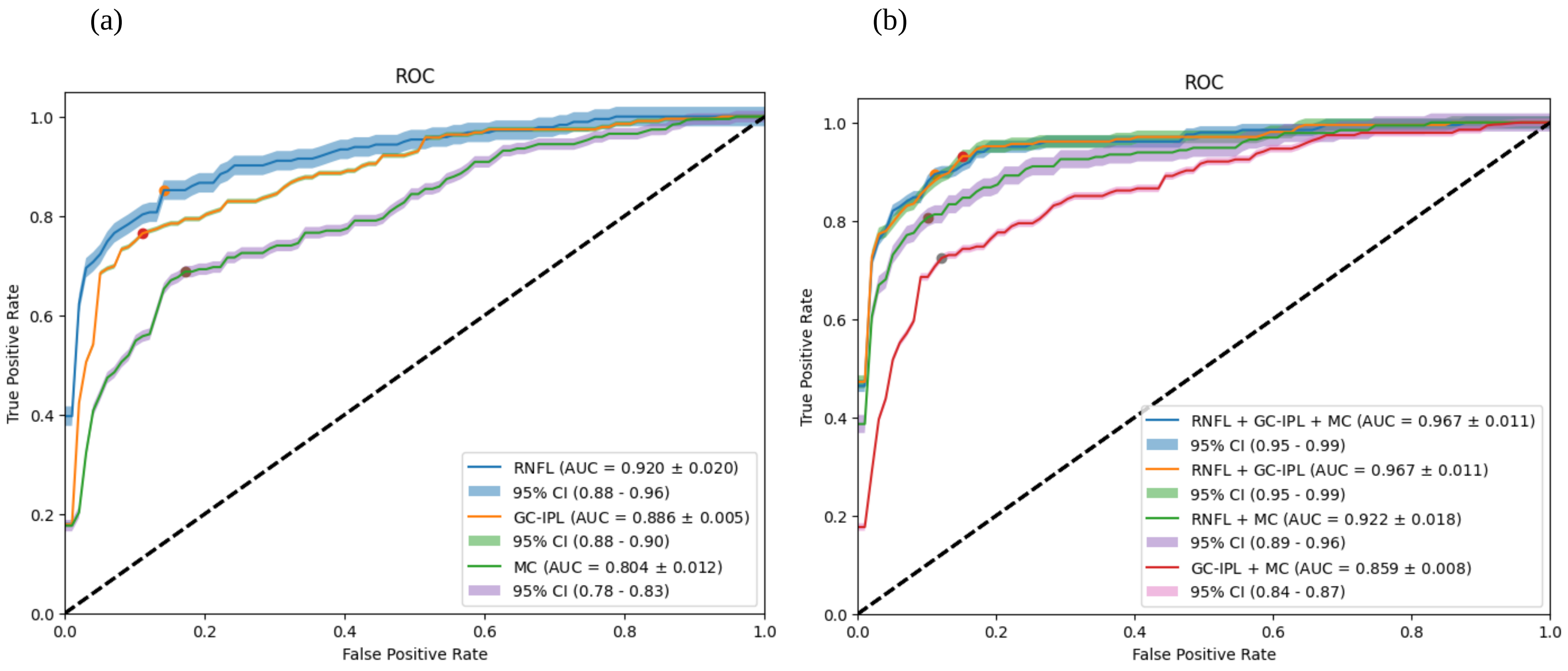


**Supplementary Figure 11**: (a) Sub-analysis using the RF classifier comparing the RNFL, GC-IPL and MC thickness features. (b) Sub-analysis using the RF classifier comparing the dual and triple combinations of the RNFL, GC-IPL and MC thickness features.

## Data Availability

The patients provided approval for data to be used as part of our study (UNSW human research ethics approval number: HC210563). Consequently, the raw data are not available for public access, but the statistically analysed OCT data in tabular format for a group of patient cohorts (normal, glaucoma/glaucoma stages) may be accessible upon reasonable request to the corresponding author.

## References


1 Danjuma KJ. Performance evaluation of machine learning algorithms in post-operative life expectancy in the lung cancer patients. *arXiv preprint arXiv:150404646* 2015.

2 Berrar D. Cross-validation. *Encyclopedia of Bioinformatics and Computational Biology* 2019; 1: 542-545.

3 Bengio Y, Grandvalet Y. No unbiased estimator of the variance of k-fold cross-validation. *Journal of machine learning research* 2004; 5: 1089-1105.

4 Reus NJ, Lemij HG, Garway-Heath DF et al. Clinical assessment of stereoscopic optic disc photographs for glaucoma: the European Optic Disc Assessment Trial. *Ophthalmology* 2010; 117: 717-723.

5 Liu S, Graham SL, Schulz A et al. A Deep Learning-Based Algorithm Identifies Glaucomatous Discs Using Monoscopic Fundus Photographs. *Ophthalmology Glaucoma* 2018; 1: 15-22.

6 Hadwin SE, Redmond T, Garway-Heath DF et al. Assessment of optic disc photographs for glaucoma by UK optometrists: the Moorfields Optic Disc Assessment Study (MODAS). *Ophthalmic and Physiological Optics* 2013; 33: 618-624.
